# CircEZH2/miR-133b/IGF2BP2 aggravates colorectal cancer progression via enhancing the stability of m^6^A-modified CREB1 mRNA

**DOI:** 10.1186/s12943-022-01608-7

**Published:** 2022-06-30

**Authors:** Bing Yao, Qinglin Zhang, Zhou Yang, Fangmei An, He Nie, Hui Wang, Cheng Yang, Jing Sun, Ke Chen, Jingwan Zhou, Bing Bai, Shouyong Gu, Wei Zhao, Qiang Zhan

**Affiliations:** 1 Departments of Gastroenterology, Wuxi People’s Hospital Affiliated to Nanjing Medical University, Department of Medical Genetics, Nanjing Medical University, Nanjing, Jiangsu Province China; 2 Department of Head and Neck Surgery, Fudan University Shanghai Cancer Center, Shanghai, China; 3 Center for Precision Medicine, Department of Laboratory Medicine, Nanjing Drum Tower Hospital, The Affiliated Hospital of Nanjing University Medical School, Nanjing, Jiangsu Province China; 4 Institute of Geriatric Medicine, Jiangsu Province Geriatric Hospital, Nanjing, Jiangsu Province China; 5 Department of Biomedical Sciences and Tung Biomedical Sciences Centre, City University of Hong Kong, Hong Kong, China; 6 Present Address: School of laboratory medicine, Chengdu medical college, Chengdu, China

**Keywords:** CircEZH2, miR-133b, IGF2BP2, CREB1, Colorectal cancer

## Abstract

**Background:**

Aberrant expression of circular RNAs (circRNAs) contributes to the initiation and progression of human malignancies, but the underlying mechanisms remain largely elusive.

**Methods:**

High-throughput sequencing was performed to screen aberrantly expressed circRNAs or miRNAs in colorectal cancer (CRC) and adjacent normal tissues. A series of gain- and loss-of-function studies were conducted to evaluate the biological behaviors of CRC cells. RNA pulldown, mass spectrometry, RIP, qRT-PCR, Western blot, luciferase reporter assays and MeRIP-seq analysis were further applied to dissect the detailed mechanisms.

**Results:**

Here, a novel circRNA named circEZH2 (hsa_circ_0006357) was screened out by RNA-seq in CRC tissues, whose expression is closely related to the clinicpathological characteristics and prognosis of CRC patients. Biologically, circEZH2 facilitates the proliferation and migration of CRC cells in vitro and in vivo. Mechanistically, circEZH2 interacts with m^6^A reader IGF2BP2 and blocks its ubiquitination-dependent degradation. Meanwhile, circEZH2 could serve as a sponge of miR-133b, resulting in the upregulation of IGF2BP2. Particularly, circEZH2/IGF2BP2 enhances the stability of CREB1 mRNA, thus aggravating CRC progression.

**Conclusions:**

Our findings not only reveal the pivotal roles of circEZH2 in modulating CRC progression, but also advocate for attenuating circEZH2/miR-133b/IGF2BP2/ CREB1 regulatory axis to combat CRC.

**Supplementary Information:**

The online version contains supplementary material available at 10.1186/s12943-022-01608-7.

## Introduction

Colorectal cancer (CRC) is the third most common malignancy and the second leading cause of cancer-related death worldwide [1]. According to the Global Cancer Statistics 2020, it is estimated that there are more than 1.9 million new cases of CRC and 935,000 deaths worldwide in 2020, representing 10% of total cancer cases and deaths [2]. Moreover, the incidence and mortality rates of CRC, especially in developing countries, are still rising rapidly. Although great progress has been made in the development of screening, diagnosis and treatment strategies, the 5-year survival rate for patients with CRC remains poor [3]. To further prolong the survival time of patients with CRC, identifying novel biomarkers towards screening, early diagnosis, prognosis and outcome evaluation of CRC therapy, and elucidating the mechanisms underlying the tumorigenesis and progression of CRC are extremely important and clinically imperative.

Circular RNAs (circRNAs) are new endogenous non-coding RNA molecules with a covalently closed loop structure, mostly being generated through a special type of alternative splicing termed backsplicing [4]. Initially, circRNAs were considered as “byproducts” or “junk” generated by abnormal splicing events [5, 6]. However, with advances in bioinformatics and high-throughput sequencing technology, a large number of circRNAs have been successfully identified in cells, tissues and organisms [7, 8]. Unlike canonical linear RNA molecules, circRNAs have no 5′ cap and a 3′ poly (A) tail, and are resistant to exonuclease digestion. Therefore, circRNAs exert unique properties across varieties of life forms due to their special covalently-closed ring structure [9].

Based on accumulating evidence, circRNAs are implicated in the development of human diseases, including diabetes, neurological disorders, cardiovascular diseases, and especially cancers [10–13]. In recent years, circRNAs are emerging as critical regulators in carcinogenesis and cancer progression by competitively sponging regulatory miRNAs or interacting with RNA-binding proteins [14, 15]. In CRC, circRNAs are aberrantly expressed and exert important effects on tumorigenesis and cancer progression [16]. For example, circPACRGL serves as a sponge for miR-142–3p/miR-506-3p to activate the expression of transforming growth factor-β1 (TGF-β1), thus promoting the aggressive phenotypes of CRC cells and providing a potentially valuable biomarker for CRC treatment [17]. Moreover, methyltransferase like 3 (METTL3)-induced circ1662 can directly bind YAP1 and aggravate CRC progression by facilitating the nuclear transport of yes-associated protein 1 (YAP1) [18]. However, most of these studies were supported by miRNAs sponge mechanisms, raising the hypothesis that hidden functions of circRNAs may exist in CRC.

In this study, circEZH2 (hsa_circ_0006357), which originated from exon 2 and exon 3 of the EZH2 gene, was screened out by RNA-seq in CRC tissues. We further demonstrated that circEZH2 significantly facilitated the proliferation and migration of CRC cells in vitro and in vivo, via the circEZH2/miR-133b/IGF2BP2/CREB1 regulatory axis. These findings not only elucidate the pathogenic mechanism of the circEZH2/miR-133b/IGF2BP2/CREB1 axis in CRC, but also provide promising therapeutic targets for patients with CRC.

## Materials and methods

### Patient tissue samples

A total of 124 CRC tissues and their adjacent normal tissues were obtained from CRC patients who underwent surgery at Wuxi People’s Hospital Affiliated to Nanjing Medical University (Wuxi, China). After surgical resection, each tumor specimen was divided into two parts. One part was immediately frozen in liquid nitrogen and stably stored at − 80 °C until RNA extraction, while the other part was fixed with formalin and sectioned, and subjected to hematoxylin and eosin (H&E) staining. Written informed consent was provided by all patients enrolled in the study. The histopathologic features of these patients with CRC were reviewed by two independent pathologists for confirmation of diagnoses. The clinical characteristics of the patient population are listed in Additional file 1: Table S1. This study was approved and monitored by the Ethics Committee of Wuxi People’s Hospital Affiliated to Nanjing Medical University (No. KY22035).

### Cell lines and cell culture

Human CRC cell lines, including COLO205 (Metastatic; Poorly differentiated), HCT15 (Well/moderately differentiated), LoVo (Metastatic; Poorly differentiated), SW620 (Metastatic; Poorly differentiated), HT-29 (Moderately differentiated) and HCT116 (Poorly differentiated), were obtained from the Cell Bank of the Chinese Academy of Sciences (Shanghai, China). COLO205, HCT15 and NCM460 (normal colon cell line) cells were cultured in Roswell Park Memorial Institute medium (RPMI-1640; Gibco). LoVo cells were cultured in Ham’s F-12 K (Kaighn’s) medium (Invitrogen). SW620 cells were cultured in Leibovitz’s L-15 medium (Gibco). HT-29 and HCT116 cells were cultured in McCOY’s 5A medium (Sigma). Normal embryonic kidney cell line HEK-293 T cells were cultured in Dulbecco’s modified Eagle’s medium (ThermoFisher). These cells were maintained in medium supplemented with 10% fetal bovine serum (Gibco) and penicillin-streptomycin (5000 U/mL) (ThermoFisher) at 37 °C in a 5% humidified CO_2_ atmosphere.

### Cell transfection

Before transfection, cells were cultured in 6-well plates and grown to approximately 60% confluence at 37 °C in a 5% humidified CO_2_ atmosphere. Chemically synthesized miRNA, miRNA inhibitor, or plasmids were transfected into cells by Lipofectamine™ 3000 transfection reagent (Invitrogen) according to Invitrogen’s instructions.

### RNA isolation and quantitative real-time PCR (qRT-PCR)

Total RNAs were isolated from tissues or cells using Trizol reagent (Invitrogen Life Technologies) according to the manufacturer’s instructions. The quantity and quality of the extracted total RNA were assessed by using a NanoDrop 2000c spectrophotometer (Thermo Scientific). For circRNA and mRNA, RNA was reverse-transcribed using HiScript III 1st Strand cDNA Synthesis Kit with gDNA Wiper (Vazyme; R312–01). AceQ Universal SYBR qPCR Master Mix (Vazyme; Q511–02) was used for the real-time qPCR analysis, with a CFX96 Touch Real-Time PCR Detection System (Bio-rad). For miRNA, reverse transcriptions were performed using miRNA 1st Strand cDNA Synthesis Kit (Vazyme; MR101–01) with specific stem-loop primers (CenePharma; China). Real-time PCR analysis was performed using miRNA Universal SYBR qPCR Master Mix (Vazyme; MQ101–01) on an ABI StepOnePlus Real-Time PCR system (Applied Biosystems). Endogenous glyceraldehyde-3-phosphate dehydrogenase (GAPDH) or small nuclear RNA (U6 snRNA) was used as an internal control, and the relative expression level for each genes was calculated by the 2^-ΔΔCt^ method. All reactions were performed in triplicate. Primer sequences are available in Additional file 2: Table S2.

### Protein extraction and Western blot

Cells were collected, washed with ice-cold phosphate buffered saline (PBS) and lysed for 30 min in RIPA buffer containing 50 mM Tris/HCl (pH 7.5), 150 mM NaCl, 1% NP40, 1% Triton X-100, 2.5 mM sodium pyrophosphate, 1 mM β-glycerophosphate, 1 mM EDTA, 1 mM Na3VO4, 1 μg/mL leupeptin. Cell lysates were centrifuged at 14,000 g for 10 min at 4 °C and the protein concentration was measured using the BCA Protein Assay kit (Pierce, Rockford, IL). The aliquots of lysates (twenty micrograms of protein) were boiled with sample loading buffer (Beyotime; P0015) for 5 min and resolved by sodium dodecyl sulfate-polyacrylamide gel electrophoresis (SDS-PAGE). After electrophoresis, proteins were electrophoretically transferred onto a polyvinylidene difluoride (PVDF; Roche) membrane by using a Semi-Dry Electroblotter (Bio-Rad). After transfer, the membrane was blocked for 2 h at room temperature in phosphate buffered saline (PBS) containing 5% (w/v) nonfat milk and 0.1% (v/v) Tween-20. The membranes were incubated with primary antibodies against human IGF2BP2 (Proteintech, 11,601–1-AP; 1: 3000), CREB1 (Proteintech, 12,208–1-AP; 1: 1000), Cyclin A2 (Proteintech, 18,202–1-AP; 1: 1000), MMP-9 (Proteintech, 10,375–2-AP; 1: 1000), GLUT3 (Proteintech, 20,403–1-AP; 1: 1000), BCL-2 (Proteintech, 12,789–1-AP; 1: 2000), GAPDH (Proteintech, 60,004–1-Ig; 1: 20000) or Hsp 70 (Proteintech, 10,995–1-AP; 1: 10000) at 4 °C overnight and followed by a 1 h incubation at room temperature with horseradish peroxidase (HRP)-linked anti-rabbit secondary antibody (Proteintech, SA00001–2; 1: 50000) or anti-mouse secondary antibody (Proteintech, SA00001–1; 1: 100000). After four washes with PBS containing 0.1% (v/v) Tween-20, immunoreactive bands were visualized by using Chemistar™ High-sig ECL Western Blotting Substrate (Tanon; 180–501).

### RNA immunoprecipitation (RIP) assay

Cells were harvested, re-suspended in 1 mL lysis buffer containing a protease inhibitor cocktail and RNase inhibitor. After centrifuging at 13,000 rpm for 10 min at 4 °C, the supernatant was incubated with 30 ~ 40 μL Protein A-Sepharose beads (Genescript) and 2 μg primary antibodies for 4 h at 4 °C, followed by washing with ice-cold 1 × PBS. The beads were incubated with Proteinase K (Sigma) using Trizol reagent (Invitrogen Life Technologies), and the purified RNA was subjected to qRT-PCR analysis.

### Pulldown assay

Biotin-labeled oligonucleotide probe of circEZH2 was commercially synthesized (RiboBio, China). Briefly, Biotin-labeled oligonucleotide probes were incubated with BeyoMag™ streptavidin magnetic beads (Beyotime; P2151) for 60 min at room temperature. After being bound to streptavidin magnetic beads, the probe-beads were incubated with whole cell lysates overnight at 4 °C. After washing with ice-cold PBS three times, miRNAs or proteins pulled down by the probed-coated beads. Subsequently, the eluted proteins were analyzed by LC-MS/MS (Shanghai Bioprofile, Shanghai, China) or Western blot.

### Cell counting Kit-8 (CCK-8) assay

Cell viability was evaluated using a Cell Counting Kit-8 (CCK-8) assay kit (Beyotime; C0037) according to the manufacturer’s instructions. Briefly, cells were seeded in 96-well plates (1000 cells/well) in the presence of medium containing 10% fetal bovine serum and penicillin-streptomycin (5000 U/mL) at 37 °C in a 5% humidified CO_2_ atmosphere. After 24 h of culture, 10 μL of CCK-8 reagent was added to each well of the 96-well plates and incubated for 2 h at 37 °C in a 5% humidified CO_2_ atmosphere. The absorbance value of each well was determined at 450 nm (reference wavelength: 650 nm) by a microplate reader (Bio-Rad).

### 5-Ethynyl-20-deoxyuridine (EdU) incorporation assay

EdU incorporation assay was performed to evaluate cell proliferation using Cell-Light EdU Apollo488 In Vitro Kit (RiboBio; C10310–3) according to the manufacturer’s instructions. Briefly, cells were cultured in 96-well plates (1000 cells/well) and grown to 40–60% confluence. Then, cells were labeled with 50 μM EdU solution for 2 h at 37 °C in a 5% humidified CO_2_ atmosphere. After washing twice with PBS, cells were fixed with 4% paraformaldehyde (PFA) for 30 min and permeabilized with 0.5% Triton X-100 for 10 min at room temperature. Apollo® fluorescent dyes were added to the cells for 30 min, and EdU-positive cells (green) were visualized under an Olympus FSX100 microscope (Olympus, Tokyo, Japan). Hoechst 33342 fluorescence was used to identify the nuclear region (blue).

### In vitro colony-formation assay

For colony-formation assays, cells were implanted into 6-well plates (1000 cells/well) and cultured in the presence of medium containing 10% fetal bovine serum and penicillin-streptomycin (5000 U/mL) at 37 °C in a 5% humidified CO_2_ atmosphere. Two weeks later, colonies were fixed in 100% methanol for 30 min, and then stained with 0.5% crystal violet solution (Beyotime; C0121; dissolved in 25% ethanol) for 30 min at room temperature. Next, the colonies were rinsed with water and the number of colonies (≥ 50 cells) was counted under a microscope (Olympus Corporation).

### Wound-healing scratch assay

Cells were cultured in 6-well plates in the presence of medium containing 10% fetal bovine serum and penicillin-streptomycin (5000 U/mL) and grown to 100% confluence at 37 °C in a 5% humidified CO_2_ atmosphere. Subsequently, wounds were manually scratched in the cell monolayer with a plastic pipette tip. Immediately after scratching, suspended cells and debris were washed twice with PBS. Then, the wounds were photographed with a wide-field optical microscope system at 0 h and 24 h. Quantitative analysis of the migration ability was calculated according to the formula: migration ability = (migration distance / scratched width) × 100%.

### Transwell migration assay

Transwell migration assay was conducted using the 8 μm pore-size transwell plates (BD Biosciences) according to the manufacturer’s instructions. Cells were plated in 300 μL of serum-free medium on upper chambers inserted into a 24-well plate (5 × 10^4^ cells/well), and bottom chambers were filled with 700 μL of medium containing 10% fetal bovine serum. After incubation for 36 h, non-migrated cells were gently removed by a cotton swab, and the cells in the lower surface of the upper chamber were fixed with 100% methanol for 30 min and stained with 0.5% crystal violet solution (Beyotime; C0121; dissolved in 75% ethanol) for 30 min at room temperature. Finally, the migrated cells were photographed and counted under a microscope (Olympus Corporation).

### Luciferase reporter assay

The sequences of circEZH2 and IGF2BP2–3’UTR and their corresponding mutation were designed, synthesized and inserted into luciferase reporter vector, including circEZH2-WT, circEZH2-Mut, IGF2BP2–3′-UTR-WT and IGF2BP2–3′-UTR-Mut, respectively. All these plasmids were co-transfected with miR-133b or inhibitor or control mimics into HCT116 and SW620 cells, respectively. Then, the relative luciferase activity was examined by Dual Luciferase Assay Kit (Promega; USA) according to the manufacturer’s protocol.

### Fluorescence in situ hybridization (FISH)

Fluorescence-labeled circRNA (Cy3) and miR-133b (FITC) probes were designed and synthesized by GenePharma (Shanghai; China). Hybridization was performed overnight with Cy3-labeled circEZH2 or FITC-labeled miR-133b probes. After being rinsed with PBS, slides were mounted with glass coverslips using Anti-fade Mounting medium (Beyotime; P0126), and the fluorescence was visualized and captured by a Leica confocal laser scanning microscope system (TCS-SP2-AOBS-MP; Heidelberg, Germany). The sequences of DNA probes for FISH were as follows:

Hsa_circEZH2: 5′-(Cy3)-CATGATTATTCTCCCTAGTCCCG-(Cy3)-3′;

Hsa_miR-133b: 5′-(FITC)-TAGCTGGTTGAAGGGGACCAAA-(FITC)-3′.

### Hematoxylin and eosin (H&E) staining

All the specimens and xenograft tissues were fixed in 10% buffered formalin, embedded in paraffin wax and sliced with a thickness of 4 μm. Then the slides were dewaxed twice in 100% xylene for 30 min at 56 °C and rehydrated through graded alcohol solutions (100, 90, 80, 70 and 50%). The slides were then rinsed in H_2_O for 5 min, stained with hematoxylin and eosin (Beyotime; C0105S) for 10 min, serially dehydrated in ethanol, transparent by 100% xylene and mounted with a coverslip using Permount (Fisher Scientific) at room temperature.

### Immunohistochemistry (IHC) staining

IHC staining was performed as previously described [19]. The H score method assigned a score of 0–300 to every CRC patient was used for the staining assessment according to the formula: H score = ΣPi (i + 1), where “i” represents an intensity score and “Pi” is the percentage of immunostained cells [20].

### In vivo assays

We chose 6-week-old female BALB/c nude mice for tumor xenograft experiments to evaluate the growth of HCT116 xenografts in vivo. Nude mice were housed in a specific pathogen-free barrier facility (12-h light/dark cycle) with ad libitum access to food and water, and maintained at a constant temperature (23 °C) and humidity (50%). HCT116 cells were then harvested by centrifugation, washed twice with 1 × PBS and suspended in ice-cold Matrigel/PBS solution (50:50 mixture; BD Bioscience) at a concentration of 2 × 10^6^ cells/100 μL. After that, cells were subcutaneously injected into the right flank of BALB/c nude mice to establish xenograft tumor models (2 million cells per mouse). The size of each xenograft was measured and recorded every four days with a digital caliper, and all the mice anesthetized and sacrificed when the subcutaneous xenografts (control group) reached a volume of 1.0 cm^3^. Tumor volume (V) was calculated using the equation: V (cm^3^) = ½ × L × W^2^, where “L” and “W” represent the length and width of the tumor mass, respectively.

To investigate tumor metastasis, approximately 1 × 10^6^ HCT116 (Control or circEZH2 KD) cells suspended in 100 μL of 1 × PBS per mouse were injected into the tail vein of male BALB/c nude mice. The IVIS Spectrum animal imaging system (PerkinElmer) was used to monitor the metastatic loci formed by HCT116 cells (40 days) with 100 μL XenoLight D-luciferin Potassium Salt (15 mg/mL; Perkin Elmer) per mouse. Mice were anesthetized and then sacrificed for tumors and metastasis which were further analyzed by H&E staining.

To evaluate the effects of circEZH2 on both initiation and regulation of colitis carcinoma, azoxymethane/dextran sodium sulphate (AOM/DSS)-induced Apc^Min/+^ mouse models were proposed. One week before experiments, adeno-associated viruses (AAV)-circEZH2 overexpression (circEZH2-OE) and AAV-control (control) were administered by enema. Apc^Min/+^ mice were intraperitoneally injected with 12 mg/kg of AOM (Sigma). After 4 days, mice were given drinking water containing 2% (w/v) DSS (MP Biomedicals, Irvine, USA) for 5 consecutive days (repeated thrice), which was then followed by 16 days of regular water. On day 60, mice were sacrificed.

BALB/c nude mice and C57BL/6 J-Apc^Min/+^ mice were purchased from the Model Animal Resource Information Platform (Nanjing, China). All animal care and experiments were performed according to the Animal Care and Use Committee Guidelines, and approved by Wuxi People’s Hospital Affiliated to Nanjing Medical University (No. 2021–012).

### Statistical analysis

Quantitative data were statistically analyzed using SPSS version 19.0 (IBM SPSS, Armonk, NY, USA) and GraphPad Prism 5.0 software (Graphpad, San Diego, CA, USA). Experimental data are presented as the mean ± standard deviation (SD) of at least three independent triplicate experiments. Student’s *t*-test was employed to determine statistical significance. Overall survival (OS) curves were calculated by Kaplan-Meier method and Log-rank test was used to determine the significance of the OS curves. Spearman’s correlation was used to analyze the relationships between groups. *P < 0.05 indicates statistically significant difference.

## Results

### Expression profiles of circRNAs in human CRC and adjacent normal tissues

**Fig. 1:**
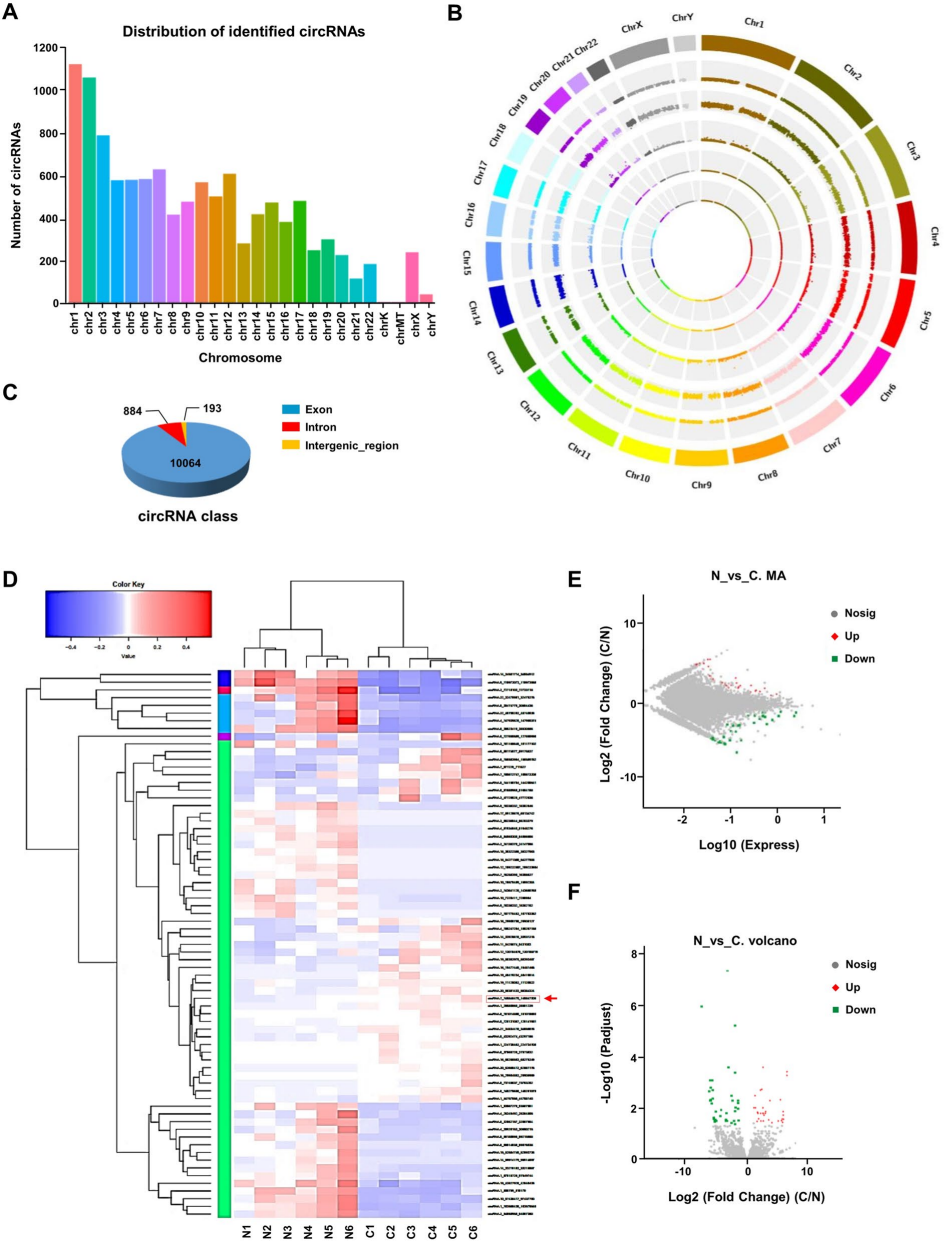
Expression profiles of circRNAs in human CRC and adjacent normal tissues. **A** Distribution of the identified circRNAs on human chromosomes. X-axis, the number of each chromosome; Y-axis, the number of circRNAs. **B** Circos plot depicting the distribution of circRNAs on human chromosomes. The outermost layer was a chromosome map of the human genome. The inner circles from outside to inside corresponded to distribution and expression of identified circRNAs on the chromosomes, distribution and expression of significantly expressed circRNAs, respectively. **C** Composition of the identified circRNAs in terms of genomic origin. **D** A cluster heap map presented the significantly dysregulated circRNAs in human CRC tissues relative to adjacent normal tissues. The red and blue strips represent high and low expression, respectively. CircEZH2 was marked in red. Scatter plot (**E**) and Volcano plot (**F**) of differentially expressed circRNAs in CRC and adjacent normal tissues

To investigate the role of circRNAs in the progression of CRC, we performed RNA-sequencing (RNA-seq) analysis of ribosomal RNA-depleted total RNA isolated from 6 paired CRC and adjacent normal tissues. We found that the host genes of these identified circRNAs were distributed in all chromosomes (Fig. 1A). Circos plot, as shown in Fig. 1B, showed the chromosome-wise location and expression of identified and expressed circRNAs on human chromosomes, and about 90.33% (10,064/11141) of circRNAs were generated from exon regions (Fig. 1C). Among the identified 71 differentially expressed circRNAs, 32 circRNAs were upregulated and 39 circRNAs were downregulated in CRC tissues relative to normal tissues (log2 fold change > 1 or < − 1; *P* < 0.05; Additional file 3: Table S3). Differentially expressed circRNAs in CRC tissues relative to matched adjacent normal tissues were visualized by hierarchical clustering (Fig. 1D), scatter plots (Fig. 1E) and volcano plots (Fig. 1F).

### Characterization of circEZH2 and its clinical significance

**Fig. 2:**
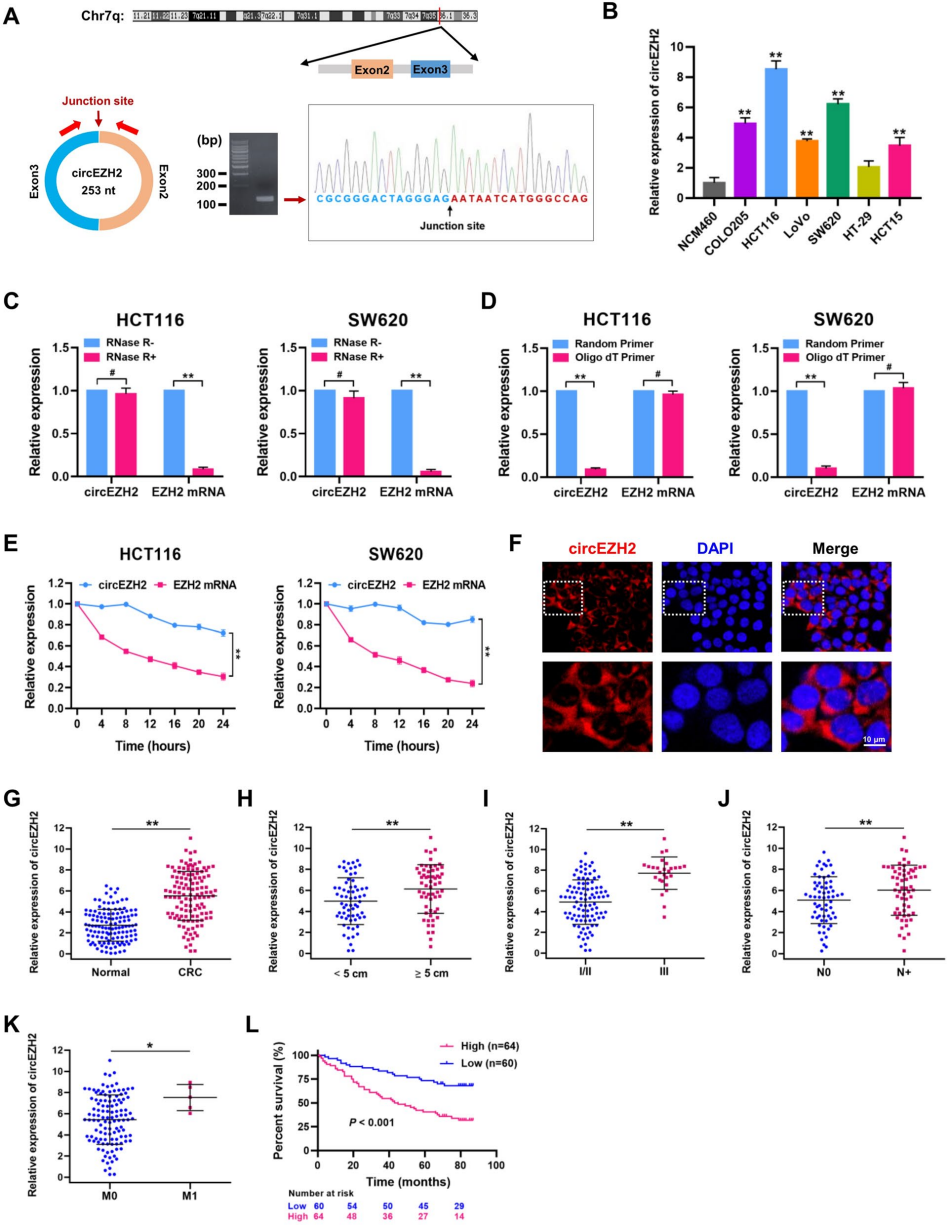
Characterization of circEZH2 and its clinical significance. **A** Schematic illustration of circEZH2 formation from EZH2 gene in chromosome 7, which was further validated by polymerase chain reaction (PCR) amplification and Sanger sequencing (right panel). **B** Relative expression of circSPON2 was determined in various human CRC cell lines (COLO205, HCT15, LoVo, SW620, HT-29 and HCT116) and normal colon cell line NCM460 by qRT-PCR assays. **C** Stability of circEZH2 and EZH2 mRNA was assessed by RNase R treatment followed by qRT-PCR in HCT116 and SW620 cells. **D** qRT-PCR analysis for the circEZH2 and EZH2 mRNA using the template cDNA reverse-transcribed by random primers and oligo dT primers using RNAs from HCT116 and SW620 cells. **E** qRT-PCR assay for the expression of circEZH2 and EZH2 mRNA in HCT116 and SW620 cells treated with Actinomycin D (2 μg/mL) at the indicated time points. **F** Fluorescence in situ hybridization (FISH) were performed with Cy3-labeled circEZH2 probes (red) to detect the location of circEZH2 in HCT116 cells. Scale bar = 10 μm. **G** Relative expression of circEZH2 in CRC tissues and matched adjacent normal tissues was analyzed by qRT-PCR assays (*n* = 124). The association between circEZH2 expression and tumor size (**H**), tumor stage (**I**), lymphatic metastasis (**J**) and distant metastasis (**K**). **L** Kaplan-Meier analysis (log-rank test) of overall survival according to circEZH2 expression level (*P* < 0.001). Data were showed as mean ± SD. *P < 0.05, **P < 0.01

Among these candidate upregulated circRNAs, hsa_circ_0006357, designated as circEZH2, attracted our attention. CircEZH2 was generated through head-to-tail splicing of exon 2 and exon 3 of EZH2 gene (253 bp), which was further validated by polymerase chain reaction (PCR) amplification and Sanger sequencing (Fig. 2A). The expression levels of circEZH2 were determined in six human CRC cell lines (COLO205, HCT15, LoVo, SW620, HT-29 and HCT116) and normal colon cell line NCM460 by qRT-PCR assay. Our results clearly showed that the expression level of circEZH2 was remarkably upregulated in these CRC cell lines compared with normal colon cell line NCM460. Notably, HCT116 and SW620 produced the highest amount of expressed circEZH2 (Fig. 2B), which were selected for further function evaluation. In HCT116 and SW620 cells, circEZH2 was observed to resist the digestion by RNase R, which specifically degraded linear RNAs but not circRNAs (Fig. 2C). Reduction in reverse-transcription efficiency by oligo-dT primers due to the lack of poly (A) tail also demonstrated the circularity of circEZH2 (Fig. 2D). Owing to its circular structure, we found that circEZH2 was more stable than linear EZH2 mRNA after Actinomycin D (a transcription inhibitor) treatment in HCT116 and SW620 cells (Fig. 2E). Fluorescence in situ hybridization (FISH) assay was used to detect the subcellular localization of circEZH2 in HCT116 cells by using Cy3-labeled probe. As shown in Fig. 2F, FISH assay revealed that circEZH2 was predominantly localized in the cytoplasm of HCT116 cells.

To validate the expression pattern and the clinical significance of circEZH2 in CRC specimens, we assembled a retrospective cohort of 124 pairs of patients diagnosed with CRC. Our qRT-PCR analyses showed that circEZH2 level in CRC tissues was conspicuously higher than that in adjacent normal tissues (Fig. 2G). High circEZH2 expression was significantly associated with tumor size, tumor stage, lymph node status and distant metastasis (Fig. 2H-K; Additional file 4: Table S4). Furthermore, Kaplan-Meier survival curves showed that higher expression of circEZH2 led to a significantly poorer overall survival (log-rank test; Fig. 2L). Thus, circEZH2 is an unfavorable circRNA for CRC.

### Knockdown of circEZH2 attenuates CRC cell proliferation and migration in vitro

**Fig. 3:**
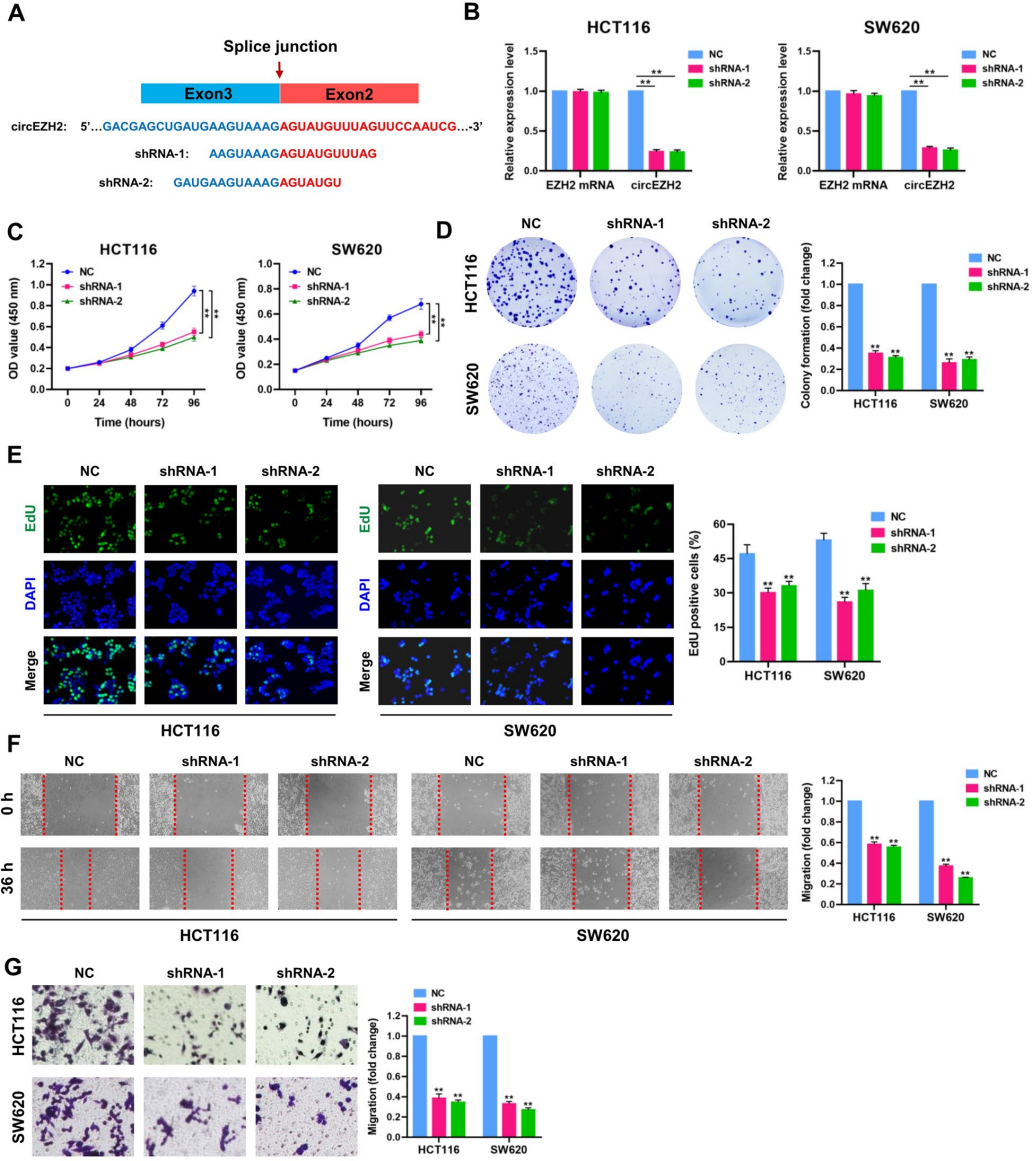
Knockdown of circEZH2 attenuates CRC cell proliferation and migration in vitro. **A** The schematic illustration of small hairpin RNAs (shRNAs) specifically targeting the back-splice junction sequences of circEZH2. **B** qRT-PCR analysis of circEZH2 and circEZH2 mRNA in HCT116 and SW620 cells treated with negative control (NC) or circEZH2 shRNAs. **C-G** CCK-8, plate colony-formation, EdU incorporation, wound-healing and transwell assays were performed to determine the proliferation and migration abilities of negative control (NC) and circEZH2-depleted (circEZH2 shRNAs) HCT116 and SW620 cells. Data were showed as mean ± SD. **P < 0.01

To evaluate the functional importance of circEZH2, we designed two shRNAs specifically targeting the back-splice sites of circEZH2 (Fig. 3A), which dramatically reduced the expression levels of circEZH2 in HCT116 and SW620 cells, but without any significant effect on the expression of linear EZH2 mRNA (Fig. 3B). The results of CCK-8 assay demonstrated that deprivation of circEZH2 markedly inhibited cell proliferation of HCT116 and SW620 cells (Fig. 3C). Colony formation assay showed that circEZH2 depletion significantly restricted the formation of colonies derived from HCT116 and SW620 cells (Fig. 3D). Consistent with expectation, EdU incorporation assay further showed that circEZH2-depleted HCT116 and SW620 cells had significantly lower percentage of EdU-positive nuclei (green) compared to control cells (Fig. 3E), indicating that circEZH2 exerts a proliferation-promoting effect in CRC cells. Subsequently, we performed wound-healing scratch assay upon knockdown of circEZH2 in HCT116 and SW620 cells. As shown in Fig. 3F, a significantly decreased migratory potential was observed in circEZH2-deficient HCT116 and SW620 cells comparing to control cells. As a further confirmation, transwell assay validated that stable knockdown of circEZH2 exerted an inhibitory effect on the migration of HCT116 and SW620 cells in vitro (Fig. 3G).

### CircEZH2 promotes colorectal tumorigenesis, growth and metastasis in vivo

**Fig. 4:**
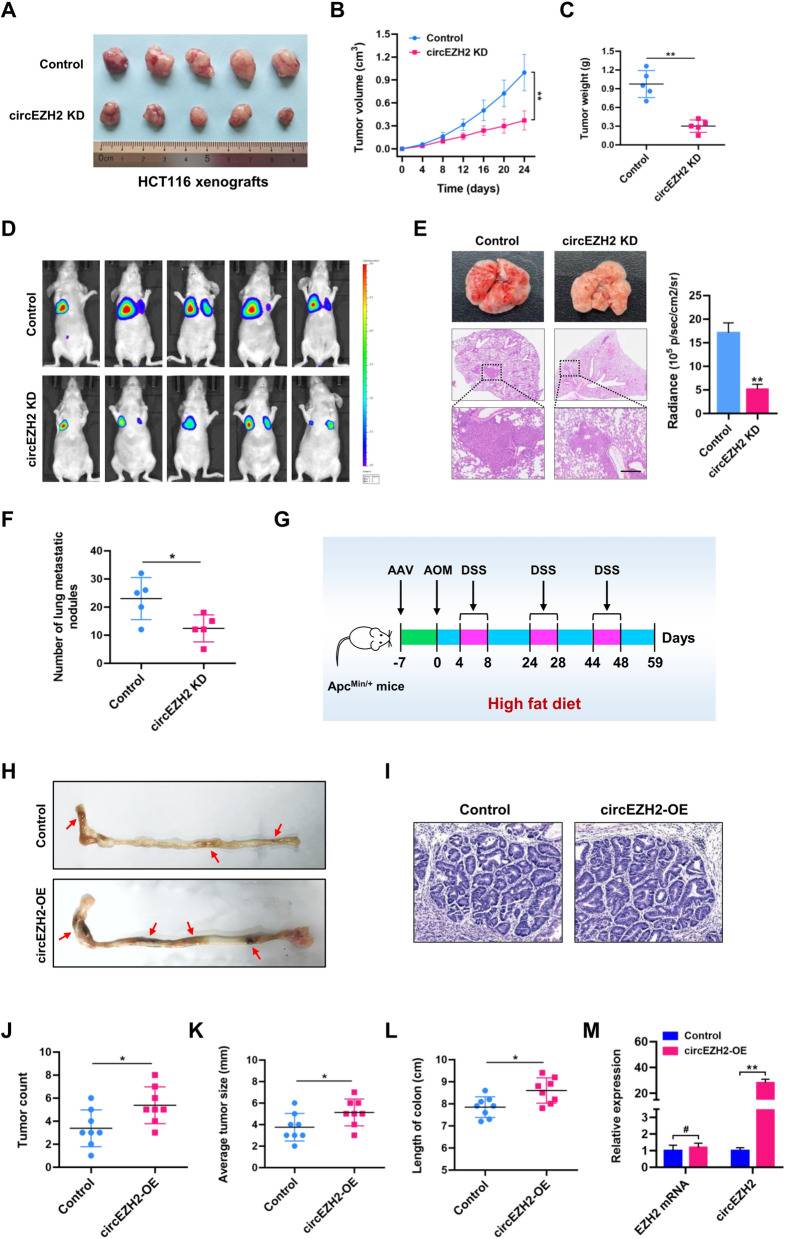
CircEZH2 promotes colorectal tumorigenesis, growth and metastasis in vivo*.* **A** Image of subcutaneous tumor xenografts in control and circEZH2 KD groups. **B** The tumor growth curves of xenografts were plotted in control and circEZH2 KD groups. **C** The tumor weights of xenografts were measured. **D** BALB/c nude mice injected with cells (control and circEZH2 KD; Five mice per group) via tail vein were imaged at 40 days by in vivo imaging system to evaluate the whole metastasis. **E** Representative images of H&E staining of lung metastasis loci. Scale bar = 200 μm. **F** The number of metastatic nodules was counted in the whole lung. **G** Scheme for the AOM/DSS-induced colon cancer model in Apc^Min/+^ mice. One week before AOM injection, AAV-control and AAV-circEZH2-OE were administered by enema. **H** Representative images of colon tissues in control and circEZH2-OE mice. Red arrows indicate visible tumors. **I** Hematoxylin and eosin (H&E) staining of colon tumors from control and circEZH2-OE mice. **J-L** The tumor number, average tumor size and length of colon in control and circEZH2-OE groups were analyzed. **M** The expression levels of EZH2 mRNA and circEZH2 in control and circEZH2-OE mice were detected by qRT-PCR assays. Data were showed as mean ± SD. ^#^ *P* > 0.05, *P < 0.05, **P < 0.01

Encouraged by the in vitro results, we further elucidated the role of circEZH2 in vivo. As shown in Fig. 4A-C, circEZH2 knockdown dramatically attenuated tumor growth in xenograft mouse models. Moreover, metastatic animal model established by tail vein injection was used to evaluate the metastasis of CRC cells in vivo. As shown in Fig. 4D and E, live imaging systems for small animals and H&E staining revealed that shRNA lentivirus-mediated knockdown of circEZH2 significantly inhibited the formation of metastatic foci in the lungs of mice in vivo. The number of metastatic nodules was significantly reduced in the lung tissues of mice when circEZH2 was knocked down (Fig. 4F). Next, we investigated whether circEZH2 is involved in CRC pathogenesis using azoxymethane/dextran sodium sulphate (AOM/DSS)-induced CRC mouse model. As shown in Fig. 4G, Apc^Min/+^ mice were treated with AOM/DSS for 59 days to induce colorectal tumorigenesis. One week before AOM injection, we used adeno-associated viruses (AAV9) to specifically overexpress circEZH2 in the colon of mice. As expected, circEZH2 infected Apc^Min/+^ mice (circEZH2-OE) developed significantly more and bigger tumors than that in control Apc^Min/+^ mice, which was histologically examined by hematoxylin and eosin (H&E) staining (Fig. 4H-J). The average tumor size and length of colon were also increased in circEZH2-OE mice (Fig. 4K and L). The qRT-PCR analysis suggested that colon tissues from circEZH2-OE mice had significantly higher expression level of circEZH2 compared to control mice, while the EZH2 mRNA was not significantly altered (Fig. 4M). Taken together, these results validate that circEZH2 facilitates colorectal tumorigenesis, growth and metastasis in vivo.

### CircEZH2 functions as a sponge of miR-133b in CRC

**Fig. 5:**
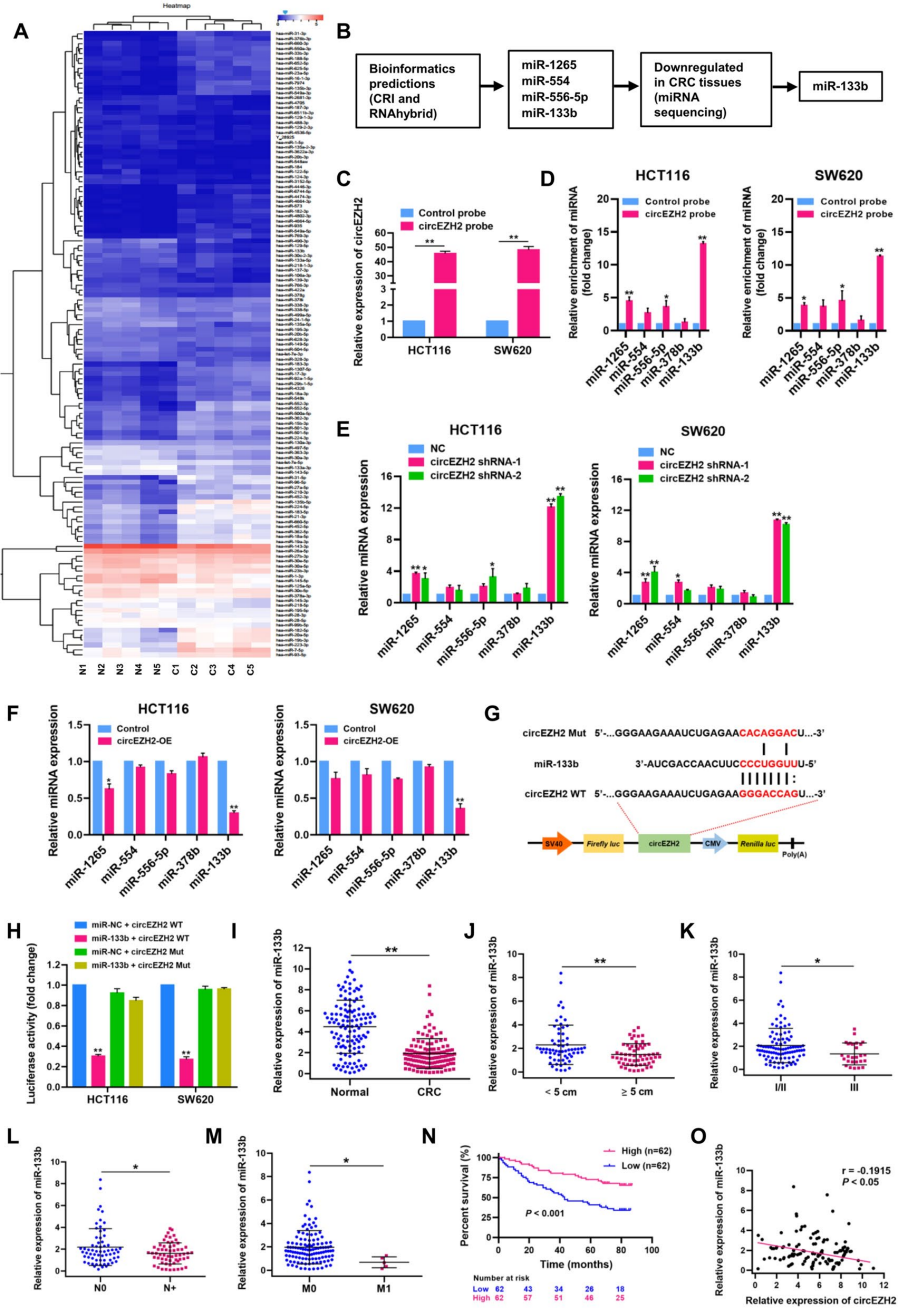
CircEZH2 functions as a sponge of miR-133b in CRC. **A** Hierarchical clustering map presented the significantly dysregulated miRNAs in human CRC tissues relative to adjacent normal tissues by high-throughput miRNA-seq. The red and blue strips represent high and low expression, respectively. **B** Schematic illustration predicting the putative miRNAs that might be sponged by circEZH2 based on miRNA-seq data and bioinformatics analyses. **C** CircEZH2 in HCT116 and SW620 cells was pulled down by a circEZH2-specific probe and determined by qRT-PCR assays. **D** Relative miRNA expression levels were determined in HCT116 and SW620 cells by qRT-PCR assays after being pulled down by circEZH2 probe or control probe. **E** Relative miRNA expression levels were determined in control and circEZH2-depleted (shRNAs) HCT116 and SW620 cells. **F** Relative miRNA expression levels were determined in control and circEZH2-OE HCT116 and SW620 cells. **G** Schematic illustration of circEZH2 wild-type (WT) and mutant (Mut) luciferase reporter vectors. **H** The relative luciferase activities were determined in HCT116 and SW620 cells after co-transfection with circEZH2 WT or Mut vectors and miR-133b mimics or miR-NC, respectively. **I** Relative expression of miR-133b in CRC tissues and matched adjacent normal tissues was analyzed by qRT-PCR assays (*n* = 124). The association between miR-133b expression and tumor size (**J**), tumor stage (**K**), lymphatic metastasis (**L**) and distant metastasis (**M**). **N** Kaplan-Meier analysis of overall survival according to miR-133b expression level (Log-rank test; *P* < 0.001). **O** Spearman’s correlation analysis revealed a negative association between miR-133b expression and circEZH2 expression in CRC tissues (r = − 0.1915; *P* < 0.05). Data were showed as mean ± SD. *P < 0.05, **P < 0.01

CircRNAs have been proven to function as competing endogenous RNAs to interact with regulatory miRNA and control miRNA expression at the post-transcriptional level. In the present study, we performed high-throughput miRNA sequencing (miRNA-seq) in five pairs of CRC tissues and matched adjacent normal tissues, and identified 140 differentially expressed miRNAs (log2 fold change > 1 or < − 1; *P* < 0.05), which were visualized by hierarchical clustering (Fig. 5A; Additional file 5: Table S5). Meanwhile, we predicted the putative miRNAs that might be sponged by circEZH2 using CRI (Circular RNA Interactome) and RNAhydbrid databases. Bioinformatics analyses revealed that four miRNAs, including miR-1265, miR-554, miR-556-5p and miRNA-133b, were potential interaction partners of circEZH2. Combining the bioinformatics prediction and miRNA-seq analyses (downregulated in CRC tissues), we selected miR-133b as the potential target of circEZH2 in CRC (Fig. 5B). RNA immunoprecipitation (RIP) was subjected to define the direct interaction between circEZH2 and miR-133b using a biotin-labeled circEZH2 probe in HCT116 and SW620 cells. Results of qRT-PCR assay suggested that circEZH2 was efficiently pulled down by biotin-labeled circEZH2 probe (Fig. 5C). Compared with other miRNAs, such as miR-1265, miR-554, miR-556-5p and miR-378b (a miRNA associated with CRC progression [21]), miR-133b was notably captured by biotin-labeled circEZH2 probe in HCT116 and SW620 cells (Fig. 5D). Furthermore, we found that circEZH2 exerted an inhibitory effect on the expression of miR-133b. CircEZH2 knockdown (shRNAs) dramatically upregulated the expression of miR-133b (Fig. 5E), while circEZH2 overexpression (circEZH2-OE) reduced the expression of miR-133b in HCT116 and SW620 cells (Fig. 5F). Interestingly, circEZH2 knockdown or overexpression did not significantly affect the expression levels of miR-1265, miR-554, miR-556-5p and miR-378b in HCT116 and SW620 cells. Next, direct interaction of circEZH2 and miR-133b was confirmed through luciferase reporter assays (Fig. 5G). Luciferase activities were significantly decreased after transfecting miR-133b mimics into HCT116 and SW620 cells, compared with that of the control group (Fig. 5H). Strikingly, miR-133b-mediated luciferase repression was significantly abolished by mutating the miR-133b binding sites in circEZH2 (circEZH2 Mut). FISH assay revealed that circEZH2 and miR-133b were co-localized in the cytoplasm of HCT116 cells (Additional file 6: Fig. S1). Collectively, our results suggested that circEZH2 functions as a sponge of miR-133b in CRC cells.

Through qRT-PCR analyses, we observed that miR-133b level in CRC tissues was significantly lower than that in adjacent normal tissues (Fig. 5I), and negatively associated with tumor size, tumor stage, lymph node status and distant metastasis (Fig. 5J-M; Additional file 7: Table S6). Kaplan-Meier curves showed that lower expression of miR-133b led to a significantly poorer overall survival (log-rank test; Fig. 5N). Furthermore, miR-133b expression was negatively correlated with circEZH2 expression in CRC tissues (Fig. 5O).

### CircEZH2 promotes CRC progression by the sponge effect towards miR-133b

**Fig. 6:**
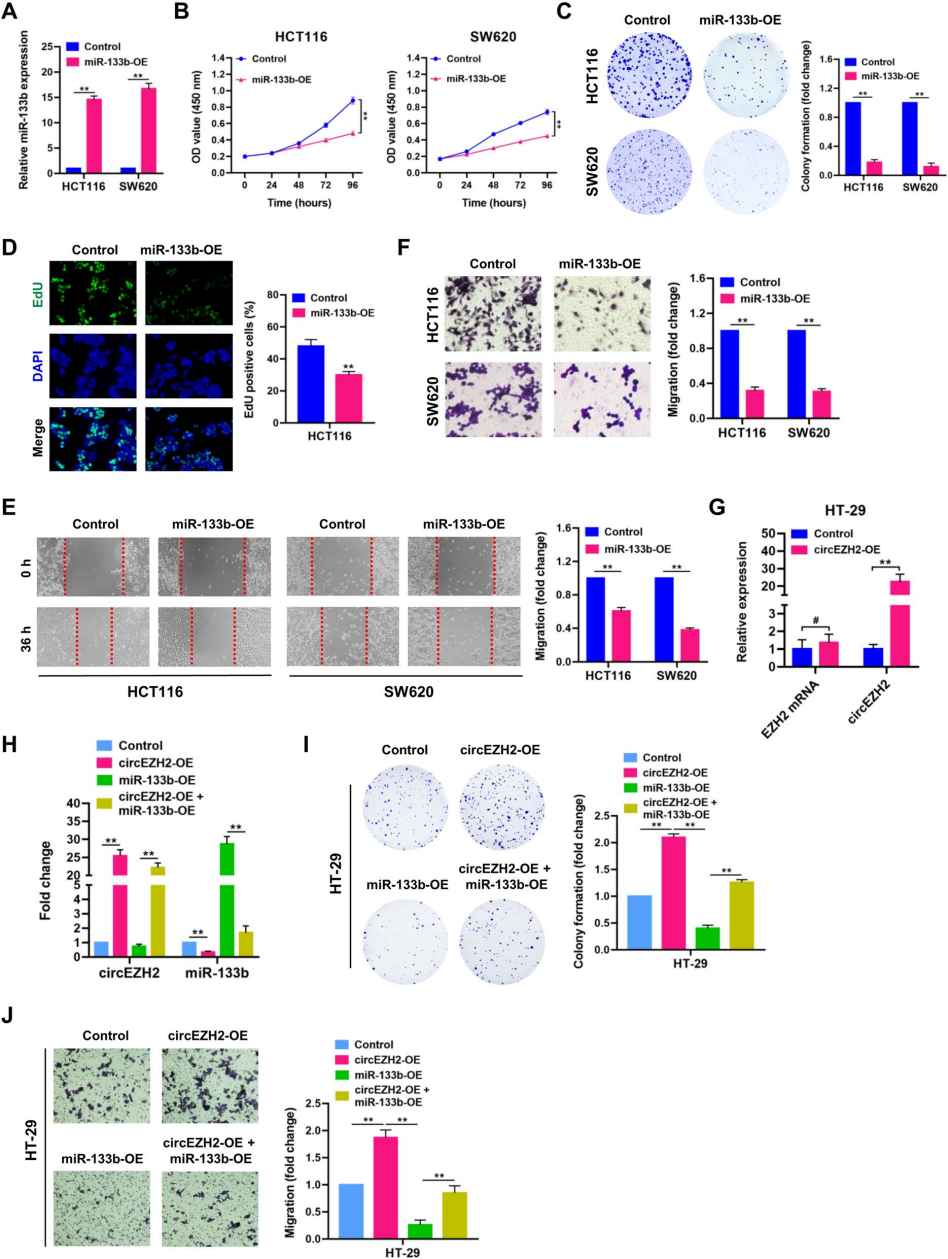
CircEZH2 promotes CRC progression by the sponge effect towards miR-133b. **A** The expression level of miR-133b in HCT116 and SW620 cells was verified by qRT-PCR. **B-F** CCK-8, plate colony-formation, EdU incorporation, wound-healing and transwell assays were performed to determine the proliferation and migration abilities of control and miR-133b-overexpressed HCT116 and SW620 cells. **G** The expression levels of EZH2 mRNA and circEZH2 in circEZH2-overexpressed HT-29 cells were verified by qRT-PCR. **H** The expression levels of circEZH2 and miR-133b in control, circEZH2-OE, miR-133b-OE and circEZH2-OE + miR-133b-OE-treated HT-29 cells were verified by qRT-PCR. **I** and **J** Colony-formation and transwell assays were performed to determine the proliferation and migration abilities of control, circEZH2-OE, miR-133b-OE and circEZH2-OE + miR-133b-OE-treated HT-29 cells. Data were showed as mean ± SD. ^#^ *P* > 0.05, **P < 0.01

To determine the roles of miR-133b in CRC cells, we established stably miR-133b-overexpressing HCT116 and SW620 cells. The expression level of miR-133b was verified by qRT-PCR assay (Fig. 6A). CCK-8, colony-formation, EdU incorporation, wound-healing and transwell assays showed that miR-133b overexpression remarkably inhibited cell proliferation and migration capacities of HCT116 and SW620 cells (Fig. 6B-F). To determine whether the tumor promoting effects of circEZH2 were exerted through its sponge effect on miR-133b, rescue experiments were performed in HT-29 cells, which has a relatively low expression of circEZH2 (Fig. 2B). Through qRT-PCR analysis, overexpression of circEZH2 was verified in HT-29 cells when circEZH2 overexpression plasmid (pCDH-CMV-circEZH2) was introduced, while the EZH2 mRNA was not upregulated significantly (Fig. 6G). The expression levels of circEZH2 and miR-133b in HT-29 cells were verified by qRT-PCR assays (Fig. 6H). Interestingly, colony-formation and transwell assays showed that circEZH2 overexpression remarkably enhanced cell proliferation and migration capacities of HT-29 cells; however, the tumor-promoting effect induced by circEZH2 was abrogated by introduction of miR-133b (Fig. 6I and J), indicating that circEZH2 promotes CRC progression by acting as a molecular sponge for miR-133b.

### CircEZH2 interacts with m^6^A reader IGF2BP2 and blocks its ubiquitination-dependent degradation

**Fig. 7:**
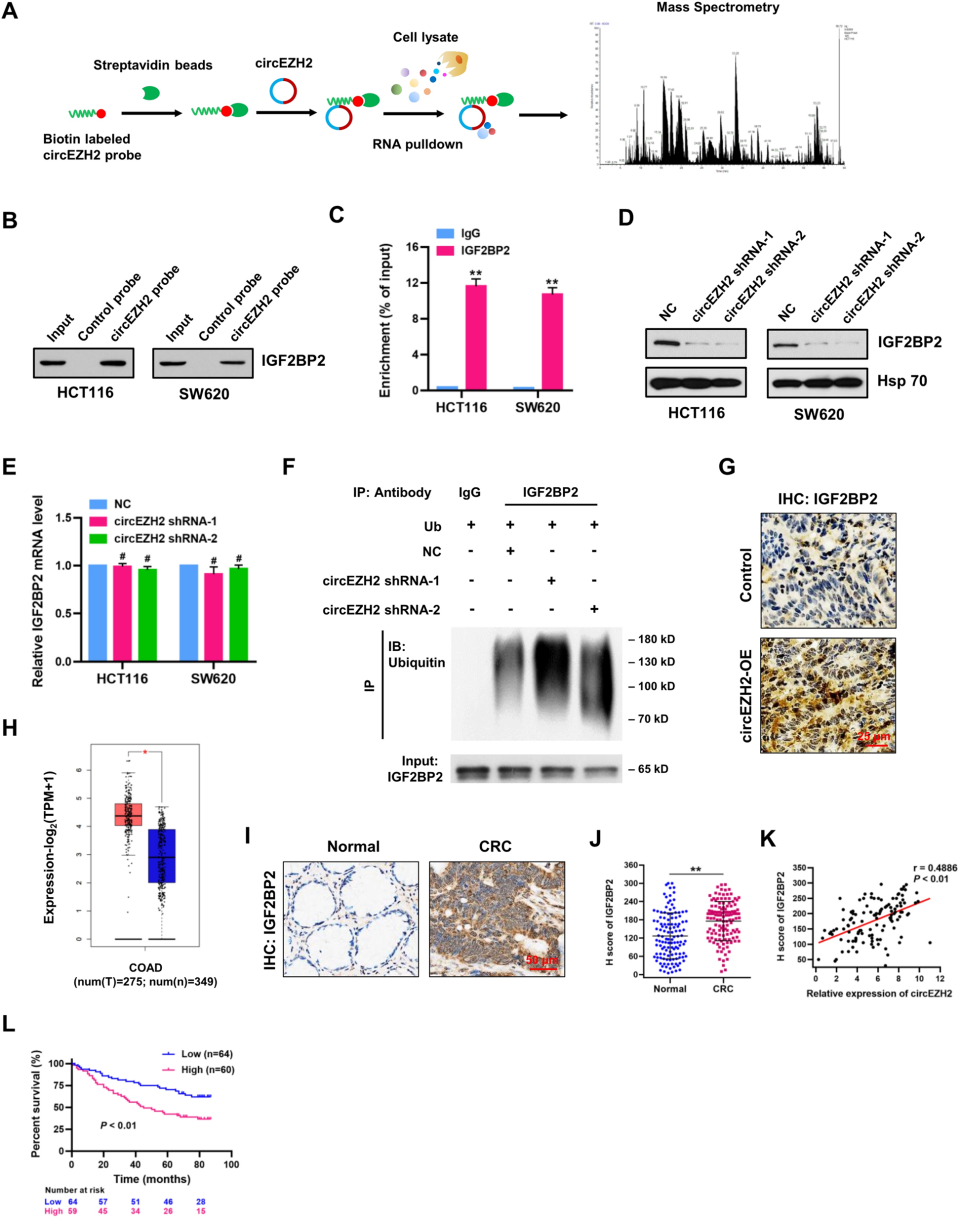
CircEZH2 interacts with m^6^A reader IGF2BP2 and blocks its ubiquitination-dependent degradation. **A** RNA pulldown was performed using biotin-labeled circEZH2 probe, followed by mass spectrometry analysis. **B** RNA pulldown assay was performed to verify the interaction between circEZH2 and IGF2BP2 in HCT116 and SW620 cells. **C** RBP immunoprecipitation (RIP) assay was performed using IGF2BP2 or IgG antibodies, followed by qRT-PCR assay for circEZH2 expression in HCT116 and SW620 cells. **D** Western blot was performed to detect the expression of IGF2BP2 in negative control (NC) and circEZH2-depleted (shRNA-1 and shRNA-2) HCT116 and SW620 cells. Hsp 70 served as a loading control. **E** qRT-PCR assay was performed to detect the mRNA levels of IGF2BP2 in negative control and circEZH2-depleted HCT116 and SW620 cells. **F** CircEZH2 knockdown accelerated the ubiquitination modification of IGF2BP2 in HCT116 cells transfected with ubiquitin (Ub) and treated with MG-132, which was detected by Western blot. **G** IHC staining of IGF2BP2 in tumors from control and circEZH2-OE AOM/DSS mice. Scale bar = 25 μm. **H** The mRNA level of IGF2BP2 in CRC (*n* = 275) and adjacent normal (*n* = 349) tissues was analyzed using TCGA database. **I** IHC staining of IGF2BP2 in CRC and adjacent normal tissues. Scale bar = 50 μm. **J** H score of IGF2BP2 in CRC and adjacent normal tissues (*n* = 124). **K** Spearman’s correlation analysis revealed a positive association between IGF2BP2 protein expression and circEZH2 expression in CRC tissues (r = 0.4886; *P* < 0.01). **L** Kaplan-Meier analysis (log-rank test) of overall survival according to IGF2BP2 expression level (*P* < 0.01). **L** Data were showed as mean ± SD. ^#^ *P* > 0.05, **P < 0.01

Multiple lines of evidence indicate that circRNAs can interact with RNA-binding proteins, which is considered an important aspect for investigating the function of circRNAs [22]. To identify the potential binding proteins of circEZH2 in CRC, we performed RNA pull-down assay with biotinylated circEZH2 probe, and followed by mass spectrometry (Fig. 7A; Additional file 8: Table S7). Among these candidate proteins interacted with circEZH2, insulin like growth factor 2 mRNA binding protein 2 (IGF2BP2) attracted our great interest because IGF2BP2 is a newly found N^6^-methyladenosine (m^6^A) “reader”. Validation experiments by RNA pulldown and Western blot showed that circEZH2 had a strong binding capability to IGF2BP2 protein in HCT116 and SW620 cells (Fig. 7B). RBP immunoprecipitation (RIP) assay revealed that circEZH2 was enriched in the anti-IGF2BP2 group, supporting the molecular interaction between circEZH2 and IGF2BP2 (Fig. 7C). Moreover, we found that circEZH2 knockdown dramatically reduced the protein levels of IGF2BP2 in HCT116 and SW620 cells (Fig. 7D), but without any significant effects on the mRNA levels of IGF2BP2 (Fig. 7E). Therefore, we speculated that circEZH2 may stabilize IGF2BP2 protein through the direct interaction. Notably, circEZH2 knockdown obviously facilitated the ubiquitination level of IGF2BP2 in HCT116 cells (Fig. 7F). Moreover, IHC staining showed that the expression of IGF2BP2 in tumors from circEZH2-OE AOM/DSS mice was higher than that from control mice (Fig. 7G). Thus, these results suggest that circEZH2 enhances the stability of IGF2BP2 protein via blocking its ubiquitination-dependent degradation.

The mRNA level of IGF2BP2 in CRC tissues was analyzed using TCGA database (Fig. 7H). We found that the mRNA level of IGF2BP2 was significantly upregulated in CRC tissues (*n* = 275) compared with that in normal (*n* = 349) tissues. Through IHC staining, we observed that IGF2BP2 was located in both cytoplasm and nucleus of tumor cells (Fig. 7I). H score analysis revealed that IGF2BP2 was significantly upregulated in CRC tissues, which was positively correlated with circEZH2 levels in CRC tissues (Fig. 7J and K). Kaplan-Meier curves showed that higher expression of IGF2BP2 led to a significantly poorer overall survival (log-rank test; Fig. 7L).

### IGF2BP2 is a direct target of miR-133b in CRC

**Fig. 8:**
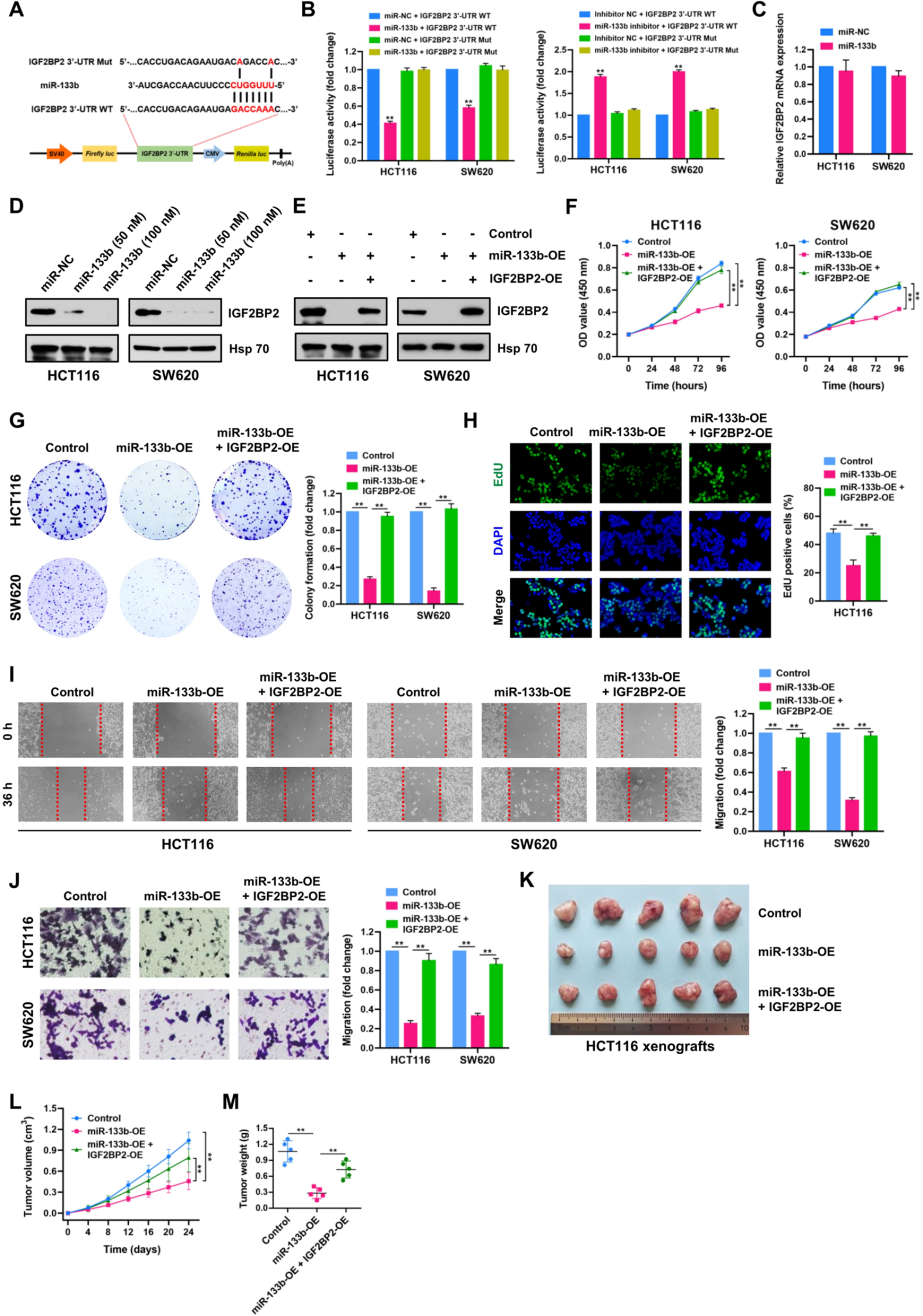
IGF2BP2 is a direct target of miR-133b in CRC. **A** Schematic illustration of IGF2BP2 3′-UTR wild-type (WT) and miR-133b binding site mutated (Mut) IGF2BP2 3′-UTR luciferases reporter vectors. **B** (Left panel) Relative luciferase activities were determined in HCT116 and SW620 cells transfected with IGF2BP2 3′-UTR WT or Mut luciferase reporter vectors and miR-133b or NC mimics. **(**Right panel**)** Relative luciferase activities were determined in HCT116 and SW620 cells transfected with IGF2BP2 3′-UTR WT or Mut luciferase reporter vectors and miR-133b inhibitor or inhibitor NC mimics. **C** IGF2BP2 mRNA expression was determined by qRT-PCR in HCT116 and SW620 cells transfected with miR-133b or NC mimics. **D** IGF2BP2 protein expression was determined by Western blot in HCT116 and SW620 cells transfected with miR-133b (50 nM and 100 nM) or NC mimics. Hsp 70 served as a loading control. **E** IGF2BP2 protein expression was determined by Western blot in control, miR-133b-OE, miR-133b-OE + IGF2BP2-OE-treated HCT116 and SW620 cells. Hsp 70 served as a loading control. **F-J** CCK-8, plate colony-formation, EdU incorporation, wound healing and transwell assays were performed to determine the proliferation and migration abilities of control, miR-133b-OE, miR-133b-OE + IGF2BP2-OE-treated HCT116 and SW620 cells. **K** Image of subcutaneous tumor xenografts in control, miR-133b-OE, miR-133b-OE + IGF2BP2-OE groups. **L** The tumor growth curves of xenografts were plotted in control, miR-133b-OE, miR-133b-OE + IGF2BP2-OE-treated HCT116 groups. **M** The tumor weights of xenografts were evaluated. Data were showed as mean ± SD. **P < 0.01

It is well established that miRNAs exert their biological functions through binding to the 3′-untranslated region (3′-UTR) of mRNA targets in a sequence-specific manner, thus facilitating translation inhibition. Coincidently, bioinformatics analysis revealed that IGF2BP2 is a putative target of miR-133b. Luciferase reporter and site mutation assays were performed to validate whether IGF2BP2 is a direct target gene of miR-133b (Fig. 8A). Our results showed that the luciferase activity of reporter plasmid carrying IGF2BP2 3′-UTR (Wild-type) was significantly decreased in miR-133b-transfected HCT116 and SW620 cells, and increased in miR-133b inhibitor group (Fig. 8B). However, these effects were almost completely abrogated when the binding sites for miR-133b were mutated (Mut). Through qRT-PCR and Western blot analyses, we found that level of IGF2BP2 protein, but not mRNA, was decreased by miR-133b in HCT116 and SW620 cells (Fig. 8C and D), indicating that IGF2BP2 acts as a direct downstream target of miR-133b in CRC.

Encouraged by this, rescue experiments were performed to explore whether miR-133b regulates CRC progression via targeting IGF2BP2. Firstly, miR-133b or IGF2BP2-overexpressed HCT116 and SW620 cells were successfully established, followed by expression analysis of IGF2BP2 using Western blot assays (Fig. 8E). Results of CCK-8, colony-formation, EdU, wound-healing and transwell assays revealed that IGF2BP2 overexpression significantly abolished the inhibitory effect of miR-133b on cell proliferation and migration of HCT116 and SW620 cells (Fig. 8F-J). Similar effects were also observed in HCT116 xenograft models (Fig. 8K-M), suggesting that the restoration of IGF2BP2 expression in CRC cells reversed the inhibition of proliferation and migration induced by miR-133b overexpression.

### CircEZH2/IGF2BP2 enhances the stability of CREB1 mRNA in CRC

**Fig. 9:**
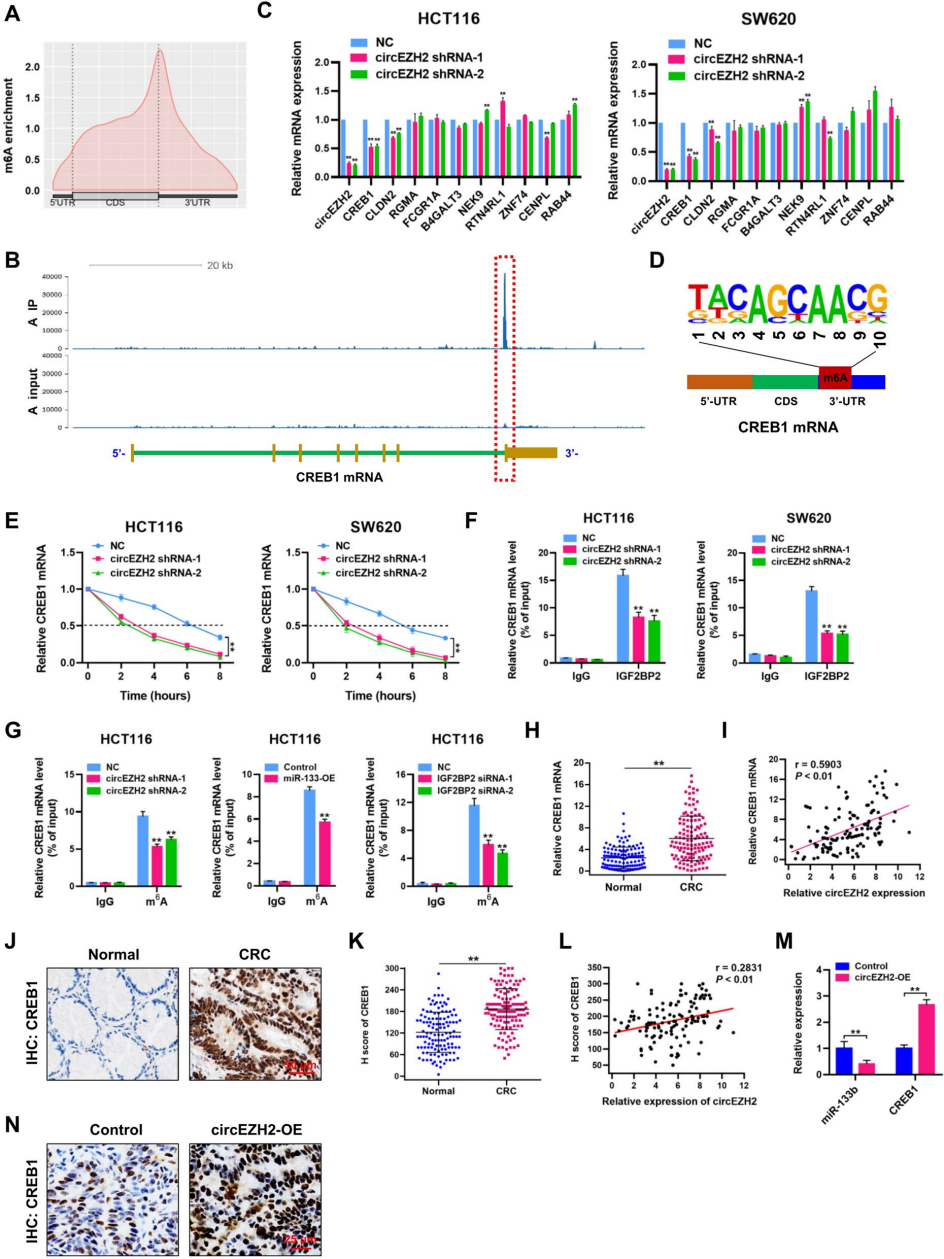
CircEZH2/IGF2BP2 enhances the stability of CREB1 mRNA in CRC. **A** MeRIP-seq (accession number: GSE179042) analysis was performed to discover the potential m^6^A modification profile in CRC tissues. m^6^A sites were displayed in 5′-UTR, CDS and 3′-UTR. **B** Schematic diagram based on MeRIP-seq showed the remarkable m^6^A modification site in the mRNA of CREB1. **C** qRT-PCR assay was performed to detect the expression of RGMA, FCGR1A, B4GALT3, NEK9, CLDN2, RTN4RL1, ZNF74, CENPL, RAB44 and CREB1 mRNAs in negative control (NC) and circEZH2-depleted HCT116 and SW620 cells. **D** Symbol showed the m^6^A motif in CRC tissues. **E** Stability of CREB1 mRNA was assessed by RNase R treatment followed by qRT-PCR in HCT116 and SW620 cells. **F** RBP immunoprecipitation (RIP) assay demonstrated the CREB1 mRNA enrichment precipitated by anti-IgG or anti-IGF2BP2 antibodies in negative control and circEZH2-depleted HCT116 and SW620 cells. **G** RIP assays demonstrated the CREB1 mRNA levels precipitated by anti-IgG or anti-m^6^A antibodies in NC and circEZH2 shRNA, control and miR-133-OE and NC and IGF2BP2 siRNA HCT116 cells. **H** Relative expression of CREB1 mRNA in CRC tissues and matched adjacent normal tissues was analyzed by qRT-PCR assays (*n* = 124). **I** Spearman’s correlation analysis revealed a positive association between CREB1 mRNA expression and circEZH2 expression in CRC tissues (r = 0.5903; *P* < 0.01). **J** IHC staining of CREB1 in CRC and adjacent normal tissues. Scale bar = 50 μm. **K** H score of CREB1 in CRC and adjacent normal tissues (*n* = 124). **L** Spearman’s correlation analysis revealed a positive association between CREB1 protein expression and circEZH2 expression in CRC tissues (r = 0.2831; *P* < 0.01). **M** The expression levels of miR-133b and CREB1 in tumors from control and circEZH2-OE AOM/DSS models were determined by qRT-PCR assay. **N** IHC staining of CREB1 in tumors from control and circEZH2-OE AOM/DSS mice. Scale bar = 25 μm. Data were showed as mean ± SD. ^#^ *P* > 0.05, **P < 0.01

IGF2BP2 is newly-established N^6^-methyladenosine (m^6^A) “reader”, which can affect the fate of mRNA in an N^6^-methyladenosine (m^6^A)-dependent manner [23]. To discover the potential m^6^A modification profile in CRC, methylated RNA immunoprecipitation (MeRIP)-seq datasets of human CRC samples (GEO accession number: GSE179042) were analyzed. MeRIP-seq analyses revealed that the m^6^A modifications mainly occur at the connection regions of 3′-UTR and CDS in CRC tissues (Fig. 9A). There were remarkable m^6^A modification sites in RGMA, FCGR1A, B4GALT3, NEK9, CLDN2, RTN4RL1, ZNF74, CENPL, RAB44 and CREB1 mRNAs (Fig. 9B; Additional file 9: Fig. S2). Moreover, qRT-PCR assay was performed to detect the expression of RGMA, FCGR1A, B4GALT3, NEK9, CLDN2, RTN4RL1, ZNF74, CENPL, RAB44 and CREB1 mRNAs in control and circEZH2-depleted HCT116 and SW620 cells. Interestingly, we found that circEZH2 loss resulted in significant downregulation of CREB1 mRNA in both HCT116 and SW620 cells (Fig. 9C). The m^6^A motif in the CREB1 mRNA was found to be GCAAC (Fig. 9D). Moreover, qRT-PCR and Western blot assays showed that the mRNA and protein expression levels of CREB1’s target genes were downregulated in circEZH2-depleted HCT116 and SW620 cells (Additional file 10: Fig. S3), including BCL-2, Cyclin A2, MMP-9 and GLUT3 [24–29]. RNA stability assays revealed that circEZH2 knockdown remarkably reduced the CREB1 mRNA stability in HCT116 and SW620 cells (Fig. 9E). RIP-qPCR assay demonstrated that CREB1 mRNA was precipitated by anti-IGF2BP2 antibody, and circEZH2 knockdown could significantly decrease the CREB1 mRNA enrichment (Fig. 9F). Further RIP-qPCR assay by using m^6^A antibody demonstrated that circEZH2 or IGF2BP2 knockdown or miR-133b overexpression resulted in significantly downregulated CREB1 mRNA levels in HCT116 cells (Fig. 9G).

In the collected CRC tissues, we found that the mRNA expression of CREB1 was much higher as compared to the adjacent normal tissues (Fig. 9H). Moreover, CREB1 mRNA level was positively correlated with circEZH2 level in CRC tissues (Fig. 9I). Through IHC staining, we found that the protein level of CREB1 was significantly upregulated in CRC tissues (Fig. 9J and K), which was positively correlated with circEZH2 level in CRC tissues (Fig. 9L). The downregulation of miR-133b and upregulation of CREB1 mRNA in tumors from circEZH2-OE AOM/DSS models were verified by qRT-PCR assay (Fig. 9M). Furthermore, the protein expression of CREB1 was obviously upregulated in tumors from circEZH2-OE AOM/DSS mice compared with control mice by IHC staining analysis (Fig. 9N). In conclusion, these results suggest that circEZH2/IGF2BP2 axis enhances the CREB1 mRNA stability.

### CircEZH2 aggravates CRC progression through modulating CREB1 expression

**Fig. 10:**
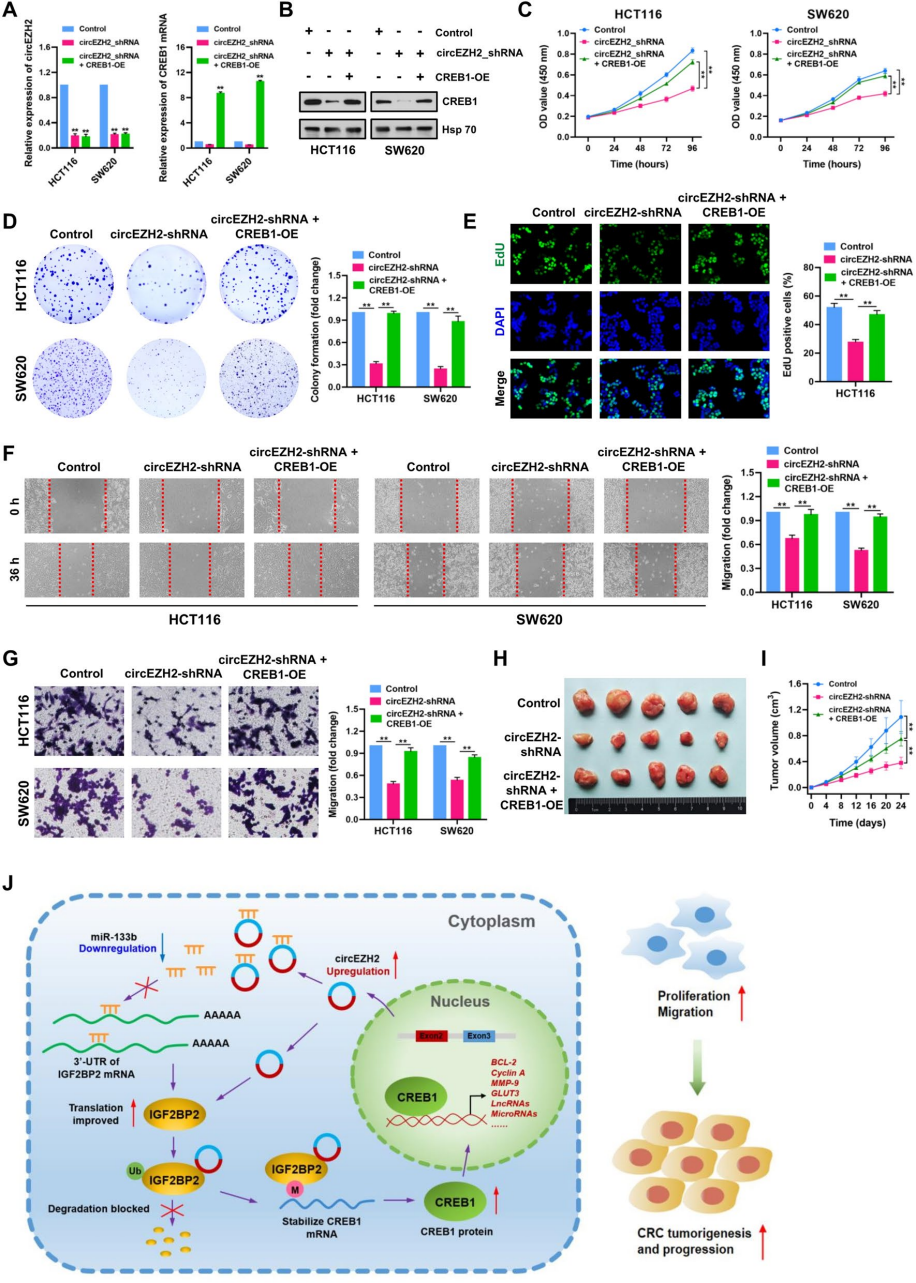
CircEZH2 aggravates CRC progression through modulating CREB1 expression. **A** CREB1 mRNA expression was determined by qRT-PCR in control, circEZH2_shRNA and circEZH2_shRNA + CREB1-OE-treated HCT116 and SW620 cells. **B** CREB1 protein expression was determined by Western blot in control, circEZH2_shRNA and circEZH2_shRNA + CREB1-OE-treated HCT116 and SW620 cells. **C-G** CCK-8, plate colony-formation, EdU incorporation, wound healing and transwell assays were performed to determine the proliferation and migration abilities of control, circEZH2_shRNA and circEZH2_shRNA + CREB1-OE-treated HCT116 and SW620 cells. **H** Image of subcutaneous tumor xenografts in control, circEZH2_shRNA and circEZH2_shRNA + CREB1-OE-treated HCT116 groups. **I** The tumor growth curves of xenografts were plotted in control, circEZH2_shRNA and circEZH2_shRNA + CREB1-OE-treated HCT116 groups. **J** Schematic illustration indicates the mechanism by which circEZH2/miR-133b/IGF2BP2/CREB1 axis aggravates CRC progression. Data were showed as mean ± SD. **P < 0.01

The above findings prompted us to evaluate whether circEZH2 can in fact drive CRC progression through modulating CREB1 expression in CRC cells. To achieve this, we successfully established control, circEZH2_shRNA, and circEZH2_shRNA + CREB1-OE-treated HCT116 and SW620 cell lines. Total RNA and protein were extracted and subjected to qRT-PCR and Western blot analyses to confirm the expression levels of circEZH2 and CREB1 (Fig. 10A and B). Functional experiments from CCK-8, colony-formation, EdU, wound-healing and transwell assays revealed that restoration of CREB1 significantly reversed the circEZH2 depletion-mediated suppression of cell proliferation and migration in CRC cells (Fig. 10C-G). Similar effects were also observed in HCT116 xenograft models, demonstrating that circEZH2 can aggravate CRC progression through modulating CREB1 expression (Fig. 10H and I).

## Discussion

CircRNAs have been discovered for more than 40 years, but circRNAs were long considered as “byproducts” or “junk” generated by abnormal splicing events with little functional potential [30]. With the advances of high-throughput RNA-sequencing and circRNAs-specific bioinformatics algorithms, massive circRNAs have been identified in cells, tissues and organisms, and some of them have been implicated in tumorigenesis and cancer progression [31, 32]. In this study, we identified a novel circRNA named circEZH2 (has_circ_0006357; 253 bp), which was generated through back-splicing of exon 2 to exon 3 of EZH2 gene. Sanger sequencing, actinomycin D and RNase R treatments were used to confirm head-to-tail junction sequences and the stability of circEZH2. The expression of circEZH2 was significantly elevated in CRC tissues and was correlated with the clinical characteristics of CRC patients. Knockdown of circEZH2 significantly inhibited the proliferation and migration of CRC cells in vitro and in vivo. CircEZH2 interacted with m^6^A reader IGF2BP2 and blocked its ubiquitination-dependent degradation. Downregulation of miR-133b, which could be sponged by circEZH2, resulted in upregulation of IGF2BP2 in CRC. Moreover, circEZH2/IGF2BP2 enhanced the stability of CREB1 mRNA in CRC and aggravated CRC progression through modulating CREB1 expression. To the best of our knowledge, our study is the first to demonstrate that circEZH2 interacted with m^6^A reader IGF2BP2 to combine with CREB1 mRNA, thereby accelerating the stability of CREB1 mRNA and driving CRC progression.

There is accumulating evidence supporting the role of circRNAs as sponges for miRNAs in modulating the expression of miRNA target genes in various malignancies [33]. Emerging evidence indicate that sponging of miRNAs is the primary approach through which circRNAs exert their biological functions. Here, combining the bioinformatics prediction and miRNA-seq profiling analyses, miR-133b was selected for further analyses. Through luciferase reporter assay, we confirmed that circEZH2 functions as a sponge of miR-133b in CRC cells. These results suggest that circEZH2 not only blocks the ubiquitination-dependent degradation of IGF2BP2 protein, but also accelerates IGF2BP2 translation via sponging miR-133b, thereby resulting in increased expression of IGF2BP2 in CRC.

Insulin-like growth factor 2 mRNA-binding protein 2 (IGF2BP2) belongs to an evolutionally conserved family of RNA-binding proteins, including IGF2BP1, IGF2BP2 and IGF2BP3 in human eukaryotic cells. IGF2BP2 dysregulation is frequently observed and plays a key tumor-promoting role in various malignancies, especially in CRC [34, 35]. However, little is known about the post-transcriptionally regulation of IGF2BP2 protein. Previous studies focus on the regulation of ubiquitination-mediated IGF2BP2 protein degradation. For example, in non-small cell lung cancer (NSCLC), circNDUFB2 could physically interact with IGF2BPs and promote ubiquitin/proteasome-mediated degradation of IGF2BPs [36]. In CRC, lncRNA LINRIS binds to the ubiquitination site of IGF2BP2, and this binding blocks IGF2BP2 degradation through the ubiquitination-autophagy pathway [37]. Given the fact that miRNAs usually have multiple downstream targets, we could not rule out the possibility that miR-133b regulates CRC progression through other potential targets. For example, Duan et al. revealed the involvement of miR-133b in the progression of human CRC via the regulation of CXCR4 expression [38]. Wang et al. showed that miR-133b plays an important role in regulating the progression of CRC through targeting HOXA9 [39], highlighting multiple functions of miR-133b in CRC.

N^6^-methyladenosine (m^6^A) is the most abundant internal RNA modification occurring in a variety of eukaryotic RNAs, including but not limited to mRNAs, tRNAs, rRNAs, and long non-coding RNAs [40]. IGF2BP2 is a newly-established as a N^6^-methyladenosine (m^6^A) “reader”, which affects the fates of mRNA in an m^6^A-dependent manner. Notably, we demonstrated that circEZH2 not only exerts its function through circEZH2-miR-133b axis, but also interacts with m^6^A reader IGF2BP2 and inhibits its ubiquitination-dependent degradation. Recent studies have shown that IGF2BP2 aggravates growth and metastasis of various types of cancer via stabilizing mRNA or translation of important modulators [23, 34, 41]. For example, circARHGAP12 interacts with IGF2BP2 and contributes the stability of FOXM1 mRNA in cervical cancer [42]. Li et al. reported that IGF2BP2 facilitates the translation elongation and mRNA stability of pyruvate dehydrogenase kinase 4 (PDK4) [43]. Moreover, circNDUFB2 promotes ubiquitination degradation of IGF2BPs through forming a TRIM25/circNDUFB2/IGF2BPs ternary complex and recruits immune cells into the tumor microenvironment by activating the RIG-1-MAVS pathway, thus attenuating tumor progression [36]. Our findings for the first time demonstrated that circEZH2/IGF2BP2 enhanced the stability of CREB1 mRNA and aggravated CRC progression through modulating CREB1 expression.

It has been well-established that cAMP response element-binding protein 1 (CREB1) belongs to a family of transcription factors whose activities are stimulated by increased intracellular cAMP [24, 27, 44–47]. Upon protein kinase A (PKA)-mediated reversible phosphorylation at serine 133 residue (Ser133ph), CREB1 can bind to DNA via a bZIP domain that recognizes cAMP response element (CRE; TGACGTCA or TGACG/CGTCA). The cAMP responsiveness of CREB1 target genes, whose promoters contain CRE, is also dependent on the presence of TATA box in their promoters [48]. Compelling evidence demonstrates that CREB1 modulates the expression of proto-oncogenes, such as Cyclin A2, EGR-1, MMP2/9, GSK3A, non-coding RNAs, etc., which are associated with cell proliferation, differentiation, apoptosis, neovascularization, inflammatory response and tumorigenesis via the ERK1/2, PKA, PKC or CaMKII signaling pathways [24–26, 28, 29, 49]. These reports strongly suggest the importance of genes constitutively regulated by CREB1 for their implicative involvement in promoting tumorigenesis and cancer progression. In this study, we demonstrated that circEZH2/IGF2BP2 enhanced CREB1 mRNA stability. In other words, circEZH2 interacts with IGF2BP2 to combine with CREB1 mRNA, forming circEZH2/IGF2BP2/CREB1 mRNA complex.

## Conclusion

These findings provide robust evidence that novel circRNA circEZH2 acts as an oncogene in CRC progression. CircEZH2 interacts with m^6^A reader IGF2BP2 and blocks its ubiquitination-dependent degradation, thereby promoting the stability of CREB1 mRNA in CRC (as illustrated in Fig. 10J). Our study expands the understanding of circRNA function in CRC pathogenesis, and the novel circEZH2/miR-133b/IGF2BP2/CREB1 axis may be a promising diagnostic marker and therapeutic target for CRC.

## Supplementary Information

**Additional file 1.**

**Additional file 2.**

**Additional file 3.**

**Additional file 4.**

**Additional file 5.**

**Additional file 6.**

**Additional file 7.**

**Additional file 8.**

**Additional file 9.**

**Additional file 10.**

## Data Availability

The datasets used in the current study are available from the corresponding author on reasonable request. The raw sequencing data in this study are available in the NCBI Sequence Read Archive (SRA) database with the BioProject accession numbers PRJNA843182 and PRJNA843315.

## Abbreviations

CRC: Colorectal cancer
circRNAs: Circular RNAs
miRNAs: microRNAs
IGF2BP2: Insulin-like growth factor 2 mRNA-binding protein 2
CREB1: Cyclic AMP-responsive element-binding protein 1
m^6^A: N^6^-methyladenosine
MeRIP: Methylated RNA immunoprecipitation
RNA-seq: RNA sequencing
CCK-8: Cell Counting Kit-8
RIP: RNA immunoprecipitation
qRT-PCR: Quantitative real-time PCR
shRNA: Short hairpin RNA
H&E: Hematoxylin and eosin
3′-UTR: 3′-untranslated region
FISH: Fluorescence in situ hybridization
RBPs: RNA binding proteins
WT: Wild type
Mut: Mutant
