# Supplementary material for: CircEZH2/miR-133b/IGF2BP2 aggravates colorectal cancer progression via enhancing the stability of m6A-modified CREB1 mRNA
Source: Mol Cancer. 2022 Jun 30;21:140. doi: 10.1186/s12943-022-01608-7 (PMC9245290; doi:10.1186/s12943-022-01608-7)
Supplement: Supplementary file 1 — Additional file 1. [file 12943_2022_1608_MOESM1_ESM.docx]

**Table S1. Clinicopathological characteristics of 124 cases of CRC patients.**

| Parameters | Number of cases (%) |
| --- | --- |
| Gender  Male  Female | 71 (57.26%)  53 (42.74%) |
| Age  ≤60  >60 | 38 (30.65%)  86 (69.35%) |
| Tumor size (cm)  ≤5  >5 | 65 (52.42%)  59 (47.58%) |
| Lymph node metastasis  N0  N+ | 65 (52.42%)  59 (47.58%) |
| Distant metastasis  M0  M1 | 119 (95.97%)  5 (4.03%) |
| TNM  I/II  III/IV | 97 (78.23%)  27 (21.77%) |
