# Supplementary material for: CircEZH2/miR-133b/IGF2BP2 aggravates colorectal cancer progression via enhancing the stability of m6A-modified CREB1 mRNA
Source: Mol Cancer. 2022 Jun 30;21:140. doi: 10.1186/s12943-022-01608-7 (PMC9245290; doi:10.1186/s12943-022-01608-7)
Supplement: Supplementary file 2 — Additional file 2. [file 12943_2022_1608_MOESM2_ESM.docx]

**Table S2. The sequences of primers used for qRT-PCR.**

| Primers | Sequence (5’-3’) |
| --- | --- |
| CircEZH2 (forward) | AACCAAGAATGGAAACAGCGAAGG |
| CircEZH2 (reverse) | AGTCGCATGTACTCTGATTTT |
| IGF2BP2 (forward) | GTTGGTGCCATCATCGGAAAGG |
| IGF2BP2 (reverse) | TGGATGGTGACAGGCTTCTCTG |
| CREB1 (forward) | GACCACTGATGGACAGCAGATC |
| CREB1 (reverse) | GAGGATGCCATAACAACTCCAGG |
| CLDN2 (forward) | GTGACAGCAGTTGGCTTCTCCA |
| CLDN2 (reverse) | GGAGATTGCACTGGATGTCACC |
| RGMA (forward) | CAACACGCCTGTGCTGCCCG |
| RGMA (reverse) | CCACCGTTCTTAGAGCCATCCA |
| FCGR1A (forward) | ATACAGGTGCCAGAGAGGTCTC |
| FCGR1A (reverse) | CCAGCTTATCCTTCCACGCATG |
| B4GALT3 (forward) | TCCTCAAGGTCTGCCCTACTGT |
| B4GALT3 (reverse) | ATTCCGCTCCACAATCTCTGCC |
| NEK9 (forward) | GGAGACAAAGCCTCCTATCGAC |
| NEK9 (reverse) | ATCCGAAGGCATAGAGCTGACC |
| RTN4RL1 (forward) | TACCTCCAGGACGACATCTTCG |
| RTN4RL1 (reverse) | GCAAAAGACGGTCCAGGTTCAC |
| ZNF74 (forward) | CGGTGAGTTTCAAGGATGTGGC |
| ZNF74 (reverse) | CACATCTGGCTTGTGCAGTGGA |
| CENPL (forward) | GGCTCAAGTGATCCTTCCACCT |
| CENPL (reverse) | AACCAGCCAGTCCACAGCACTT |
| RAB44 (forward) | AGAGATTCGGCTGTTGCTCCTG |
| RAB44 (reverse) | TGGTCCTCTGTGTCGTTGCTGT |
| BCL-2 (forward) | GGTGGGGTCATGTGTGTGG |
| BCL-2 (reverse) | CGGTTCAGGTACTCAGTCATCC |
| Cyclin A2 (forward) | CGCTGGCGGTACTGAAGTC |
| Cyclin A2 (reverse) | GAGGAACGGTGACATGCTCAT |
| MMP-9 (forward) | TGTACCGCTATGGTTACACTCG |
| MMP-9 (reverse) | GGCAGGGACAGTTGCTTCT |
| GLUT3 (forward) | GCTGGGCATCGTTGTTGGA |
| GLUT3 (reverse) | GCACTTTGTAGGATAGCAGGAAG |
| GAPDH (forward) | GGAGCGAGATCCCTCCAAAAT |
| GAPDH (reverse) | GGCTGTTGTCATACTTCTCATGG |
