# Supplementary material for: CircEZH2/miR-133b/IGF2BP2 aggravates colorectal cancer progression via enhancing the stability of m6A-modified CREB1 mRNA
Source: Mol Cancer. 2022 Jun 30;21:140. doi: 10.1186/s12943-022-01608-7 (PMC9245290; doi:10.1186/s12943-022-01608-7)
Supplement: Supplementary file 4 — Additional file 4. [file 12943_2022_1608_MOESM4_ESM.docx]

**Table S4. Association between circEZH2 and clinicopathological features of CRC patients.**

| Parameters | CircEZH2 expression | *P* value* |
| --- | --- | --- |
| Gender  Male  Female | 5.63±2.48  5.38±2.12 | *0.2779* |
| Age  ≤60  >60 | 5.67±2.61  5.46±2.21 | *0.3225* |
| Tumor size (cm)  ≤5  >5 | 4.98±2.23  6.13±2.31 | ***0.0027**** |
| Lymph node metastasis  N0  N+ | 5.08±2.22  6.02±2.38 | ***0.0122**** |
| Distant metastasis  M0  M1 | 5.44±2.33  7.52±1.24 | ***0.0249**** |
| Tumor stage  I/II  III | 4.39±2.26  7.70±1.56 | ***0.0001**** |

**P* < 0.05 is considered significant.
