# Supplementary material for: CircEZH2/miR-133b/IGF2BP2 aggravates colorectal cancer progression via enhancing the stability of m6A-modified CREB1 mRNA
Source: Mol Cancer. 2022 Jun 30;21:140. doi: 10.1186/s12943-022-01608-7 (PMC9245290; doi:10.1186/s12943-022-01608-7)
Supplement: Supplementary file 6 — Additional file 6. [file 12943_2022_1608_MOESM6_ESM.docx]

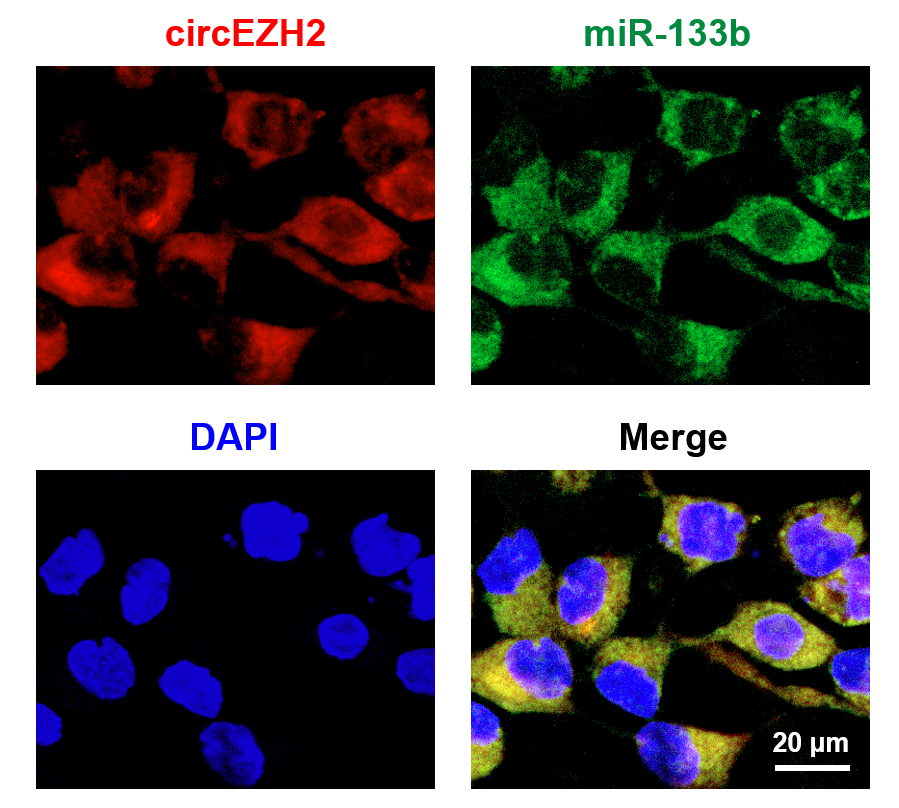


**Figure S1**. FISH assay was performed with Cy3-labeled circEZH2 (red) and FITC-labeled miR-133b probes (green) to detect the location of circEZH2 and miR-133b in HCT116 cells. Scale bar = 20 μm.
