# Supplementary material for: CircEZH2/miR-133b/IGF2BP2 aggravates colorectal cancer progression via enhancing the stability of m6A-modified CREB1 mRNA
Source: Mol Cancer. 2022 Jun 30;21:140. doi: 10.1186/s12943-022-01608-7 (PMC9245290; doi:10.1186/s12943-022-01608-7)
Supplement: Supplementary file 7 — Additional file 7. [file 12943_2022_1608_MOESM7_ESM.docx]

**Table S6. Association between miR-133b and clinicopathological features of CRC patients.**

| Parameters | CircEZH2 expression | *P* value* |
| --- | --- | --- |
| Gender  Male  Female | 1.94±1.54  1.88±1.25 | *0.7861* |
| Age  ≤60  >60 | 1.95±1.29  1.91±1.48 | *0.8747* |
| Tumor size (cm)  ≤5  >5 | 2.30±1.67  1.48±0.92 | ***0.0011**** |
| Lymph node metastasis  N0  N+ | 2.19±1.69  1.61±0.97 | ***0.0231**** |
| Distant metastasis  M0  M1 | 1.97±1.42  0.68±0.47 | ***0.0473**** |
| Tumor stage  I/II  III | 2.07±1.49  1.35±0.95 | ***0.0******18**** |

**P* < 0.05 is considered significant.
