# Supplementary material for: CircEZH2/miR-133b/IGF2BP2 aggravates colorectal cancer progression via enhancing the stability of m6A-modified CREB1 mRNA
Source: Mol Cancer. 2022 Jun 30;21:140. doi: 10.1186/s12943-022-01608-7 (PMC9245290; doi:10.1186/s12943-022-01608-7)
Supplement: Supplementary file 9 — Additional file 9. [file 12943_2022_1608_MOESM9_ESM.docx]

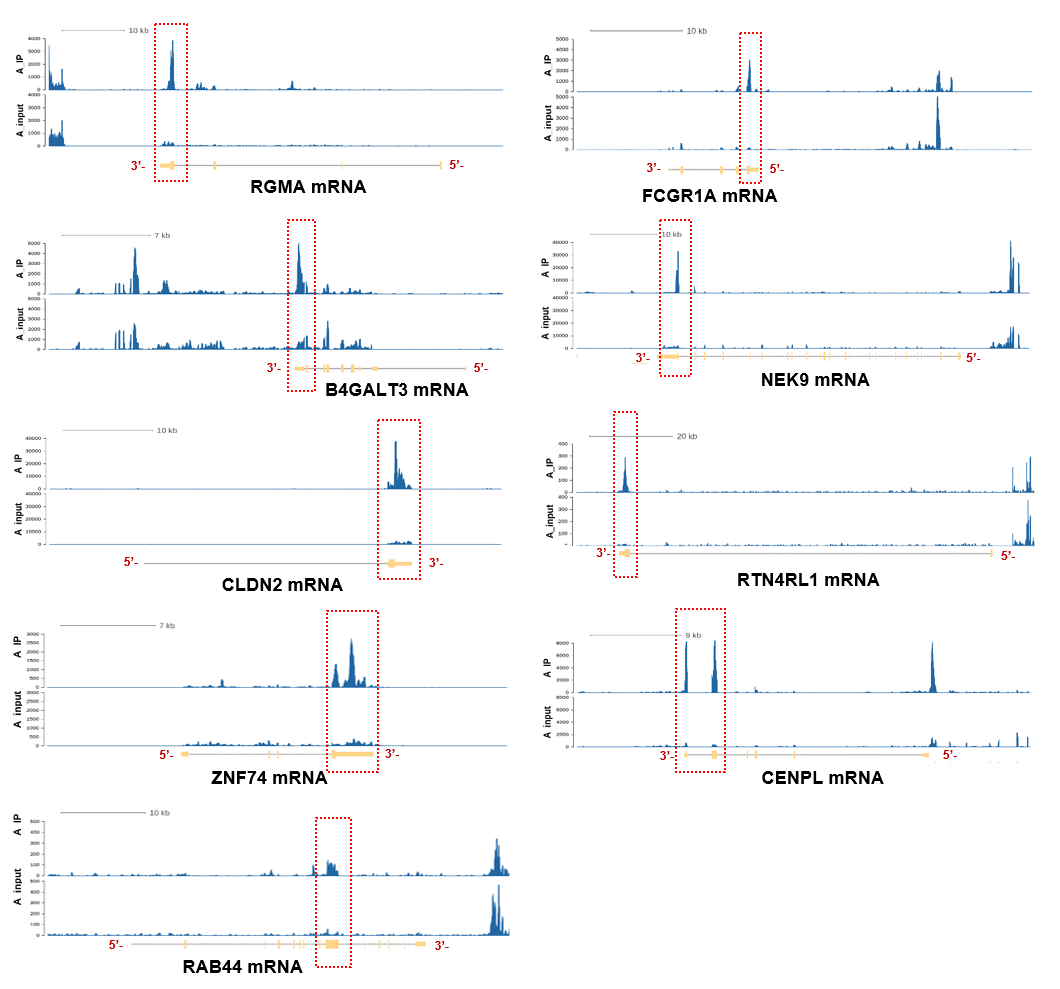


**Figure S2**. Schematic diagram based on MeRIP-seq (accession number: GSE179042) showed the remarkable m^6^A modification site in the RGMA, FCGR1A, B4GALT3, NEK9, CLDN2, RTN4RL1, ZNF74, CENPL and RAB44 mRNAs.
