# Supplementary material for: CircEZH2/miR-133b/IGF2BP2 aggravates colorectal cancer progression via enhancing the stability of m6A-modified CREB1 mRNA
Source: Mol Cancer. 2022 Jun 30;21:140. doi: 10.1186/s12943-022-01608-7 (PMC9245290; doi:10.1186/s12943-022-01608-7)
Supplement: Supplementary file 10 — Additional file 10. [file 12943_2022_1608_MOESM10_ESM.docx]

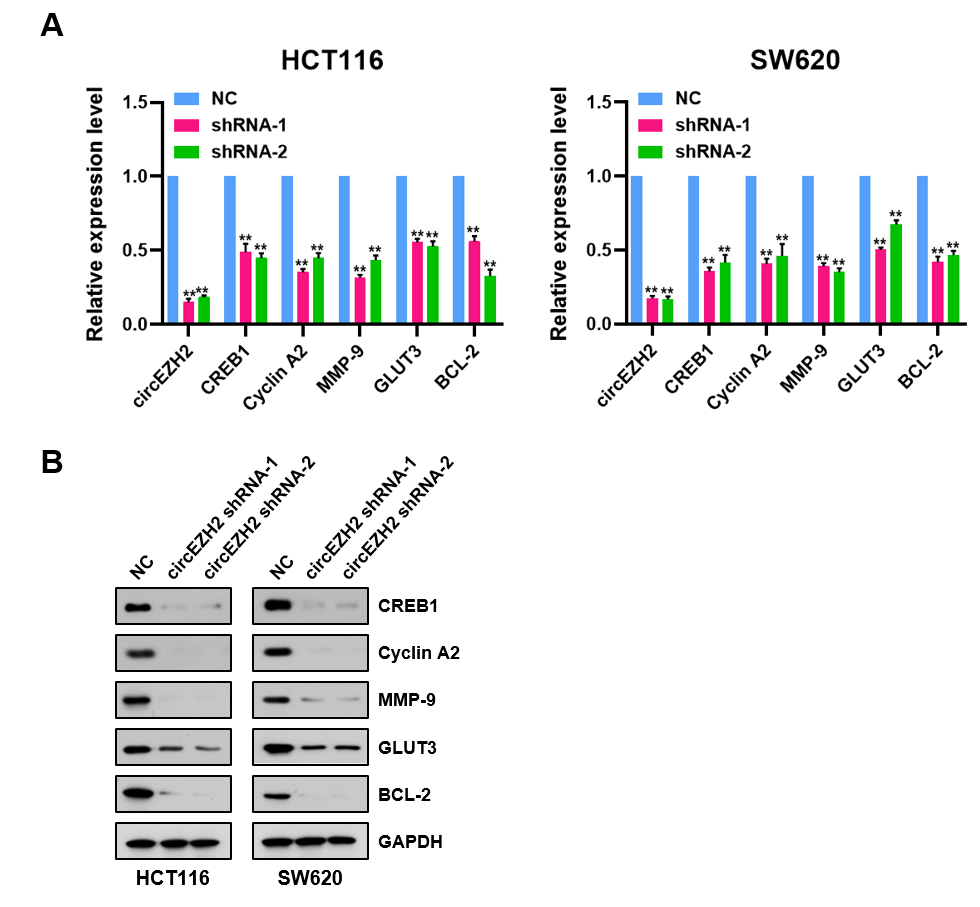


**Figure S3**. Relative expression levels of CREB1’s target genes in control and circEZH2-depleted HCT116 and SW620 cells were determined by qRT-PCR and Western blot assays. GAPDH was used as a loading control.
